# Effect of Ceria Addition to Na_2_O-ZrO_2_ Catalytic Mixtures on Lignin Waste Ex-Situ Pyrolysis

**DOI:** 10.3390/molecules26040827

**Published:** 2021-02-05

**Authors:** Adam Yeardley, Giuseppe Bagnato, Aimaro Sanna

**Affiliations:** 1 Advanced Biofuels Lab, Institute of Mechanical, Process and Energy Engineering, School of Engineering and Physical Sciences, Heriot-Watt University, Edinburgh EH14 4AS, UK; AJY1@hw.ac.uk; 2 School of Chemistry and Chemical Engineering, Queen’s University Belfast, David Keir Building, 39-123 Stranmillis Rd, Belfast BT9 5AG, UK; G.Bagnato@qub.ac.uk

**Keywords:** pyrolysis, lignin, bioethanol waste, catalysis, metal oxides, phenol, ZrO_2_, ceria

## Abstract

Waste lignin is a potential source of renewable fuels and other chemical precursors under catalytic pyrolysis. For this purpose, four mixed metal oxide catalytic mixtures (Cat) derived from Na_2_CO_3_, CeO_2_ and ZrO_2_ were synthesised in varying compositions and utilised in a fixed bed reactor for catalytic vapour upgrading of Etek lignin pyrolysis products at 600 °C. The catalytic mixtures were analysed and characterised using XRD analysis, whilst pyrolysis products were analysed for distribution of products using FTIR, GC-MS and EA. Substantial phenolic content (20 wt%) was obtained when using equimolar catalytic mixture A (Cat_A), however the majority of these phenols were guaiacol derivatives, suggesting the catalytic mixture employed did not favour deep demethoxylation. Despite this, addition of 40–50% ceria to NaZrO_2_ resulted in a remarkable reduction of coke to 4 wt%, compared to ~9 wt% of NaZrO_2_. CeO_2_ content higher than 50% favoured the increase in conversion of the holo-cellulose fraction, enriching the bio-oil in aldehydes, ketones and cyclopentanones. Of the catalytic mixtures studied, equimolar metal oxides content (Cat_A) appears to showcase the optimal characteristics for phenolics production and coking reduction.

## 1. Introduction

Industry is being driven evermore towards the use of renewable, environmentally benign feedstocks as the push to further sustainable development increases in pace, in line with the seventh principle of green chemistry [1]. This is largely motivated by a requirement to rapidly decarbonise and reduce environmental impact to create a sustainable future in mitigating climate change. One such method for achieving these goals may lie in the use of abundant secondary biomass, namely lignocellulosic residues from biorefineries as a feedstock, which are a potentially rich source of chemical precursors, such as phenol and furfural. 

Phenol is a value-added chemical component used in the polymer, pharmaceutical and dye industries as a feedstock. Key examples of valuable phenol derivates may generally be described as alkyl-phenols used as monomers for plastics production and more specifically compounds such as hydroquinone and salicylic acid as pharmaceutical reagents. 

The state-of-the-art processing route to produce phenol is via the cumene process, at times licensed as the Hock process, during which cumene is oxidised in several stages and then cleaved to form phenol and acetone [2]. Hence, the natural motivation in this case is to search for alternative production methods of the value-adding chemicals to further sustainable development of these industries. Lignocellulosic lignin derived from woody residues is a prime candidate, as any carbon emissions related to the process could offset by the carbon sequestration properties of the forest sources. The structure of lignin is that of a phenylpropanoid type polymer [3], of which it is constituents are *p*-hydroxyphenyl (H), guaiacyl (G) and syringyl (S) units. Lignin can be largely isolated from woody components into commercially derived products such as Etek (hydrolytic type) lignin from Sekab’s bioethanol production process [4]. Uniquely, due to the treatment process, the lignin itself is high in derived sugars and related products [5], which makes it suitable for pyrolysis to generate bio-oil, while makes it less attractive for production of pure lignin due to the structural modifications and rearrange on the surface of the lignocellulose when compared to organosolv methods [6]. Therefore, the composition of the raw feedstock will have a strong impact on the composition of lignin and especially the option to separate the extractives and other lignocellulosic compounds from lignin and all that will impact the gas and bio-oil composition [7].

Pyrolysis is an important step of most thermochemical processes that aims to densify biomass, simultaneously reducing its high O content. The conditions applied largely influence the solid, liquid and gas yields of the resulting bio-product [7]. Since pyrolysis is not able to completely remove O, it is typically followed by hydrodeoxygenation (HDO), which minimise O content in presence of H_2_ and catalysts [8]. Growing research is focusing on hydropyrolysis, also known as H_2_-aided pyrolysis, with the aim to maximise organics recovery in the bio-oil fraction and minimise coking in the HDO step [9]. This is mostly due to the success of the ex-situ IH2^®^ process that Shell Catalysts and Technologies licensed from GTI and is testing at demonstration scale (5 t/day) in India [10]. For example, the ex-situ hydropyrolysis of beech wood was studied in 26 bar hydrogen in a fluid bed reactor operated at 450 °C with several different catalysts, followed by HDO in fixed bed (370–400 °C) using a sulphided commercial NiMo/Al_2_O_3_ catalyst. Their results show that the best performing catalyst (HYCP) gave a condensable organic yield of 25 wt% daf corresponding to the highest obtained energy recovery of 58% and reduced coke on the spent catalyst to ~20 wt% [9]. 

Pyrolysis of biomass and its components has been well studied, paying particular attention to application of catalysts by Direct Deoxygenation (DDO), in order to selectively upgrade the pyrolysis vapour products into bio-oils of desired compositions and reduce O content to minimise H_2_ consumption in the following HDO step [11,12,13,14]. Other proposed reactions pathways include alkylation (AL), demethoxylation (DMO), oxidation (OX), demethylation (DME) and hydrogenation (HYD) among others [15,16,17,18]. 

The pyrolysis of biomasses of varying structures showed that catalytic cellulose pyrolysis produced 50–70% oxygenates (including all oxygenated aromatics except for phenols classified separately), whilst lignin pyrolysis produced 60–80% phenolics showing a clear advantage for using lignin-rich biomass for phenolics [12]. The pyrolysis of Etek type lignin was compared to that of lignins derived from different hydrolysis processes resulting in the lowest char yield (~22%), due to its intrinsic high content in holocellulose, suggesting it is a good feedstock for pyrolysis [3]. 

It is well known that DDO by catalytic pyrolysis in presence of zeolites is particularly attractive due to the selective production of aromatics but has the drawback of resulting in severe deactivation by coking and excessive carbon losses in gas phase. Metal oxides are also widely investigated with the purpose to increase the yield of desired products by first combining small oxygenates into big ones within transportation fuel range due to their superior ability to ketonisation and aldol condensation and then undergoing HDO to remove oxygen [19]. For example, CaO can effectively reduce acids, anhydrosugars and phenols, increasing the formation of hydrocarbons and cyclopentanones, while metal oxides such as ZrO_2_ and Zr_0.5_Ce_0.5_O_2_ can efficiently promote the conversion of light oxygenates to big molecules through ketonisation and/or aldol-condensation [19]. The use of ZrO_2_-TiO_2_ for the catalytic upgrading of pyrolysis vapours concerning a lignin model compound, guaiacol, resulted in the increase in phenol yields (21.4%) when using guaiacol as a feedstock [13]. The impact on pyrolysis of cellulose and lignin of the addition of alkali metals such as Na to ZrO_2_ was also studied indicating that the resulting Na/ZrO_2_ promoted decomposition of cellulose favouring hydrogen production derived from cracking of pyrolysis derivatives, while, in the case of lignin, it resulted in the largest combined yield of monomeric phenolics (17.5 wt%) and alkylphenols (6 wt%) (compared to Ce, NiCe and MgCe addition), which was linked to the mild basicity of Na/ZrO_2_ [17,18]. Addition of Ce to ZrO_2_ instead resulted in increase in bio-oil yield [18]. Furthermore, the oxidative depolymerisation of prot and alkali lignin in the presence of cobalt impregnated ZrO_2_ catalysts at 140 °C selectively produced (67 area%) guaiacol monomers [15]. These studies suggest that the mixture of basic metal oxides and ZrO_2_ is a good support for lignin depolymerisation. 

The literature shows that catalysts for biomass pyrolysis still suffer from coking, so that it is essential to develop coke resistant materials with the ability to retain organics in the bio-oil fraction. Since previous work showed that Na/ZrO_2_ is a good catalyst for recovering phenols in bio-oil and ceria is resistant to coking, for this study, a combination of metal oxides of sodium, cerium and zirconium has been selected due to the potential synergies in enhancing the bio-oil yield and phenolics recovery and reducing coke formation in the catalytic pyrolysis of Etek lignin. To do so, an ex-situ bench scale configuration (where the biomass and the upgrading catalyst are not in contact) was selected for the catalytic pyrolysis, as it is considered a fast and reliable way to evaluate catalysts’ performance [19]. The catalysts studied were then characterised using XRD, and the bio-oil analysed using GC-MS and FTIR. Cat_A with equimolar composition resulted in a remarkable reduction of coke to 4 wt% and a propensity to recover phenols from Etek lignin.

## 2. Results

### 2.1. Catalytic Mixtures Performance

Catalytic mixtures’ performances with regards to products distribution are shown in Table 1. Etek lignin pyrolysis in absence of catalyst was taken as reference to establish the catalytic mixtures influence to generate condensable bio-oil [20]. A recent work where NaZrO_2_ was used for the ex-situ pyrolysis of the same waste lignin was used as reference to understand the effect of ceria addition on the products distribution [18]. 

Cat_A presented equal stoichiometric quantities of the constituent metal oxides. This catalytic mixture resulted in an adequate conversion of Lignin (71%), however a high proportion of char (29 wt%) and a bio-oil yield of 36 wt% were produced. Catalytic Mixture B (Cat_B) consisted of a higher stoichiometric proportion of sodium oxide (Na:Ce ratio of 2:1) compared to Cat_A. The results show a lower bio-oil yield (33 wt%) and larger non-condensable gases (NCGs) yield compared to Cat_A. Catalytic Mixture C (Cat_C) utilised a higher stoichiometric proportion of cerium oxide. This mixture showed the highest performances in terms of bio-oil yield (44 wt%), which largely appeared to have been offset from the gaseous products (26 wt%). Such a significant increase in bio-oil yield can be therefore linked to CeO_2_ mild basicity when compared to the two catalytic mixtures rich in sodium and to its ketonisation activity. Catalytic Mixture D (Cat_D) utilised a higher stoichiometric proportion of zirconium and exhibited unfavourable qualities with regards to product phase distributions, with gases at 43 wt%, and only 30 wt% of the starting lignin waste was recovered in bio-oil.

Overall, it can be noticed that the oil yield decreases when Na is more abundant in the mixtures and that the simultaneous addition of ceria seems to promote recovery of organics in the bio-oil fraction. The use of metal oxides catalysts such as Zr_0.5_Ce_0.5_O_2_ can effectively promote the conversion of light oxygenates (e.g., from holo-cellulose) to big molecules through ketonisation and aldol-condensation, resulting in large bio-oil yield [19].

Relative to the literature studies undertaken, as shown in Table 1, it may be observed that the yield of bio-oil is lower compared to uncatalysed pyrolysis, denoting the cracking activity of the catalytic mixtures. Direct comparison to Na_2_ZrO_3_ catalysed Etek lignin shows largely consistent results in terms of conversion due to the fact that an ex-situ configuration was used, and the char yield is therefore not affected by the catalyst presence [18]. However, bio-oil yield is not aligned possibly due to the relative difference in the experimental set-up. From the comparison of the four catalytic mixtures it can be concluded that the predominant factor impacting conversion favouring bio-oil is the increased quantity of cerium oxide, while the sodium oxide favours NCGs due to its enhanced basicity vs. ceria. The reduced bio-oil yield of Cat_A and Cat_B vs. the previously tested NaZrO_2_ could be linked to the different mineral phases present. 

**Figure 1:**
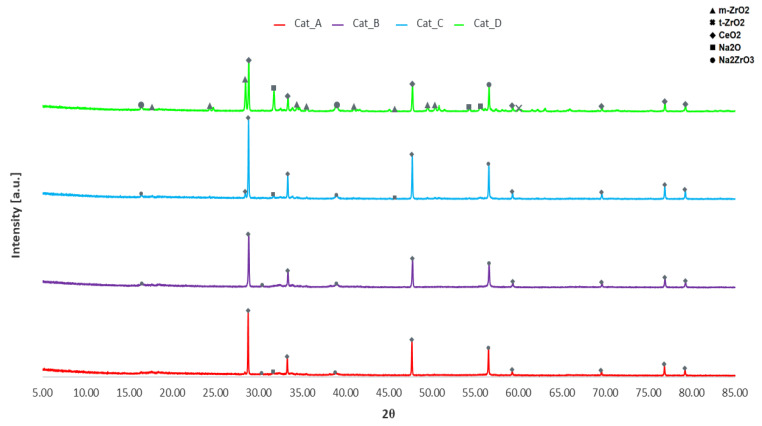
XRD patterns of Catalytic Mixtures A–D.

XRD analysis was performed to determine the phases of metal oxides present in the calcined catalytic mixtures (see Figure 1). XRD patterns of the individual phases are clearly evident, suggesting the powder is simply a mixture of metal oxide powders in crystalline phase. Notably, Na_2_O is present in addition to the desired Na_2_ZrO_3_ with prominent peaks in Cat_D at approximately 31.7° and 56.5°, respectively. It may therefore be hypothesised that not much of the Na_2_O reacted with ZrO_2_ during calcination to be converted to Na_2_ZrO_3_. Two distinct phases for ZrO_2_ are monoclinic and tetragonal with only traces of the tetragonal phase. However, both phases appear to be under-represented in XRD results, only being prominent in Cat_D, which has a higher molar proportion of ZrO_2_ in the initial mixture. Of note is the absence of peaks for Na_2_CO_3_, of which the largest peaks at 35.9° and 39.3° would be observed. Therefore, Cat_A and Cat_B mineral phases differ from the previously developed NaZrO_2_, since the latter was mainly made of sodium zirconate phases and this could explain the difference denoted in bio-oil yield.

### 2.2. Bio-Oil Characterisation

#### 2.2.1. GC-MS Analysis

**Figure 2:**
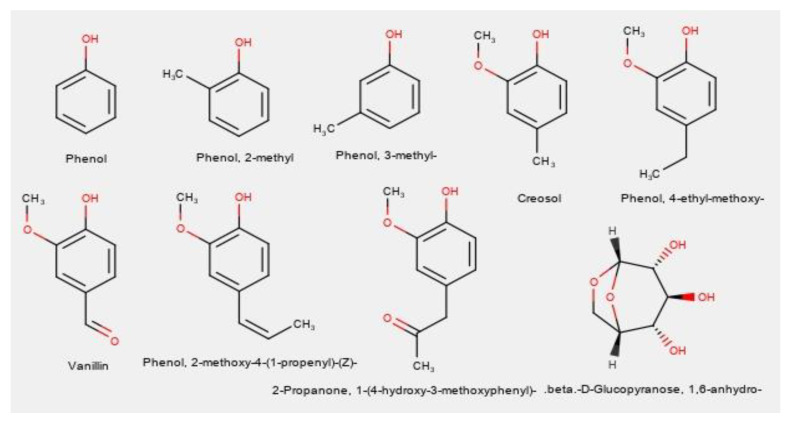
GC-MS determined common compounds from catalytic Etek lignin pyrolysis.

**Figure 3:**
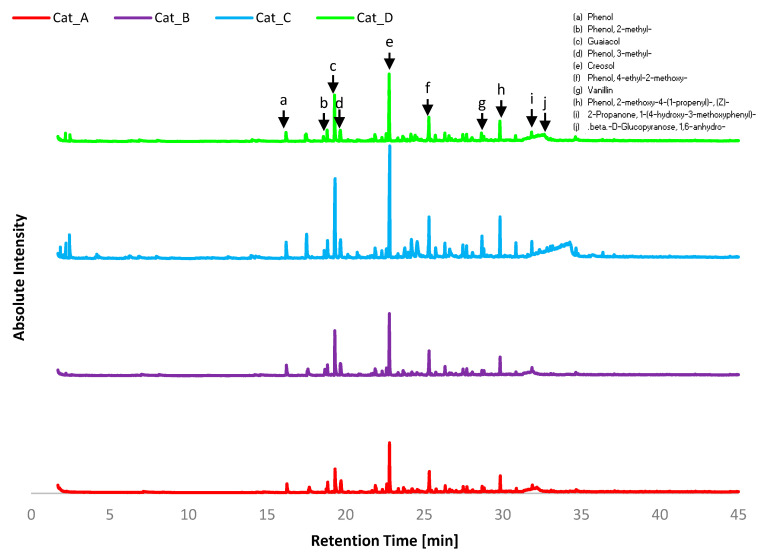
GC-MS chromatograms for bio-oil from catalytic pyrolysis using Catalytic Mixtures A–D. Common compounds are listed as a–j.

Gas Chromatography–Mass Spectrometry was performed for the bio-oils produced using the four catalytic mixtures. The structures of the main identified compounds are given in Figure 2, where it can be observed that eight of these compounds are phenol derivatives, of which five compounds also have methoxy groups, indicating guaiacol derivatives. Figure 3 shows instead the GC-MS chromatograms for each catalytic mixture. 

Of the twenty most abundance compounds across all four bio-oil product samples, half were listed as common compounds a–j. However, the abundance of each of these compounds was not equivalent across each sample, suggesting there is indeed preferential selectivity towards specific products from catalytic vapour upgrading. In has to be noted that compounds with volatilisation temperature higher than 300 °C were not detectable by GC-MS. 

Compounds were categorised based upon their functional groups as can be seen in Table 2. Vanillin, as an example, was classified as an aldehyde as this is an aldehyde derivate of guaiacol. Overall, the bio-oils produced using the catalytic mixtures pyrolysis were enriched in phenols and reduced quantities of other functional groups, such as aldehydes, ketones and cyclopentanones, with the latest functionalities mostly deriving from the holocellulose in Etek lignin.

Equimolar Cat_A had a majority yield of phenols and subsequent derivatives (largely guaiacols) at 55.5% yield, whilst hydrocarbons was the next most abundant group at 8.4%. Undesired functionalities such as acids represented only 2%. In comparison, Lu et al., who conducted pyrolysis across a wide range of catalysts using milled wood lignin (poplar wood), found that Pd/CeTiO_2_ (Rutile) resulted in 37.2% phenols and 9.5% acids [21]. This difference can be linked to the basicity of the catalytic mixtures. The presence of aldehydes and ketones suggests sugars functionalisation during the pyrolysis vapour upgrading. The high content of alcohols and hydrocarbons suggests further reactions such as the reduction of aromatic aldehydes and ketones to aromatic alcohols may have occurred. Compounds of interest for general comparison are PAHs, as their low content suggests low polymerisation of aromatics occurred.

Cat_B yielded 67.7% phenols, significantly more than the other mixtures, suggestive of selective pyrolysis and demethoxylation of the abundance guaiacol monolignols. This is highly indicative of the increased sodium content (~30 wt%) favouring more homogenous phenols rather than highly functionalised derivative. This is also confirmed by the abundancy (77%) of phenols when only Na_2_ZrO_3_ was used under similar conditions [18]. Bio-oil from Cat_B shows an overall significant decrease in most functional groups, the exceptions being PAHs, which are shown to increase to 6.7%. Aldehydes and ketones may be considered to lessen the quality of the bio-oil produced, due to their known instability and tendency to react with phenols forming polymeric resins. In this case, Cat_B lessen both functionalities.

Cat_C differs most significantly from other bio-oil products in the range of component functional groups as well as having a wider range of products overall, as can be seen in the GC-MS chromatograms (Figure 3). Comparing Cat_C results to those of Cat_A (1:1:1) shows decreased phenol content 41.2% vs. 55.5% and abundance of aldehydes and ketones making up 7.4% and 11.8% of reaction products, respectively, suggesting the cracking into condensable compounds of the holocellulose fraction in Etek lignin. A significant change in CPO is observed with 15.9% yield, the highest of all cases. Under pyrolysis conditions, ceria-based catalysts were effective in the conversion of hydroxy-carbonyls and anhydrosugars into ketones, cyclopentanone and 2-cyclopentenones, by selective C–O cleavage and C–C bond formation [22]. This was related to enhanced reduction of CeO_2_ under pyrolysis conditions, where the catalyst is likely to have a rich concentration of Ce^3+^. The addition of a strong base (e.g., CaO) to CeO_2_ led to enhanced yields of C_6_/C_7_ ketones and 2-cyclopentenone, indicating aldol condensation reactions increased [22]. In our study, the catalytic mixtures that presented the larger CeO_2_ content resulted in an increased content of cyclopentenones, while abundance of Na_2_O did not promote recovery of CPO from the holocellulose fraction. Further rearrangement of sugars to derivative sugars is also evident concerning Cat_C at 5.2%, which is over 3% higher than in the bio-oil from pyrolysis from any other mixture. Once again suggesting CeO_2_ content favours different reaction pathways, rearranging the volatiles into ketones and cyclopentanones by ketonisation and Piancarelli rearrangement. 

Comparison was made via adapting the GC-MS data from [18,20] for analysis with the same rules applied as to the data obtained in this study. Overall, fewer compounds were generated, with less variation between types of compounds, particularly with an absence of acids, PAHs and cyclopentanones, suggesting these are a direct product of catalytic pyrolysis of the organic vapours. 

Further in-depth analysis was conducted with regards to categorising the phenols present (see Table 3), which were divided into phenol (entirely unsubstituted), guaiacols, catechols, methyl phenols, alkyl phenols and others. The distinction was made between methyl phenols (including ortho, meta and para cresols) and alkyl phenols in that any alkyl groups beyond methyl groups is classified as an alkyl group. Further substitutions such as nitro, alkyl alcohol, ketone and aldehyde groups were classified as others. Further classification in this manner allows for analysis of the interactions that are likely to have taken place regarding the methoxy group of the dominant guaiacol compounds.

Overall, the total phenolic compounds present largely similar subtypes of phenols, namely various alkyl and methoxy-substituted groups. When comparing the quantities of phenol itself, the yield does not vary significantly, with only Cat_C varying to below 4.0%. Guaiacols however vary significantly, with the highest ZrO_2_ content Cat_D yielding the highest guaiacols content, with the high proportion of guaiacols indicating that demethoxylation does not occur to a high degree in the absence of marked basicity. The presence of such high guaiacol contents and relatively low proportions of other subtypes of phenols in comparison to the other catalysts is highly suggestive that ZrO_2_ does not promote further reactions involving the methoxy group but does however promote substitution on the aromatic ring. Increased catechols content is observed when considering Cat_C.

Methyl phenols content is at the lowest when considering Cat_C, however proportions of catechols and alkyl phenols are increased. In comparison to Cat_C and previously reported NaZrO_2_ catalysts [18], the lowest guaiacols (58.9%) and a higher methyl phenols content (23.5% or 4.7 wt% based on starting Etek lignin) is found when Cat_A is used, which shows a greater preference towards demethoxylation, which can be considered higher quality bio-oils due to the decrease of guaiacols. However, total phenolics (19.8 wt%) and alkyl phenols (2.2 wt%) were found to be much higher using NaZrO_2_ (28.7 wt% and 11.2 wt%) [18].

#### 2.2.2. FTIR Analysis

**Figure 4:**
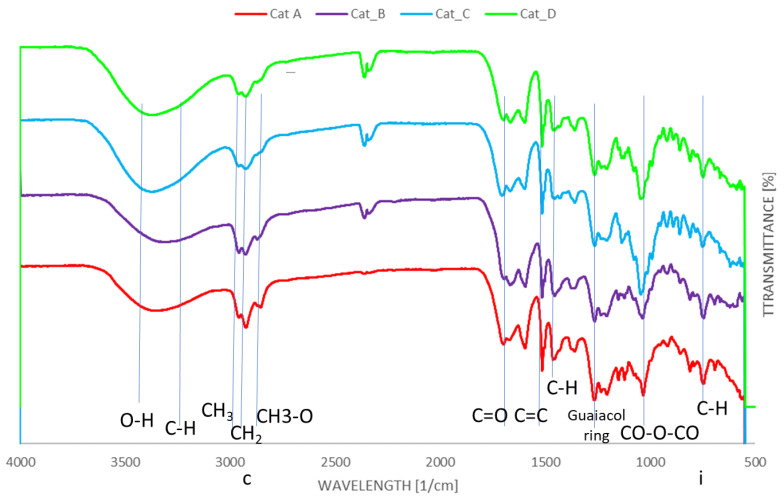
FTIR spectra for the bio-oils produced by using the four catalytic mixtures.

Fourier transform infrared spectroscopy of bio-oils and spent catalytic mixtures were carried out to investigate the differences in main functional groups present in the bio-oils. For all FTIR studies, Sigma-Aldrich FTIR tables were used for identification [23]. Figure 4 reports the FTIR analysis for the bio-oils. The broad absorption band within 3650–3400 cm^−1^ is attributed to OH groups, including OH stretch of phenols (3650–3530 cm^−1^), while the band between 3000 and 3400 cm^−1^ is assigned to weak C−H stretching vibration of aromatic groups [24,25]. The four catalytic mixtures showed absorption bands of C-H stretching in -CH_2_- and -CH_3_ group at 2960–2933 and 2853 cm^−1^ and C=O stretching of carbonyl functionality (1694–1701 cm^−1^) [6]. It may be noted that significantly more easily distinguishable absorption bands are present when considering Cat_C and Cat_D, indicating a wider array of functional groups as the GC-MS results suggest. The C=O stretching can be related to cyclopentanones shown to be present from GC-MS traces. This band was observed to be relatively broader in Cat_C traces, which was indicated by GC-MS data to have a higher yield of cyclopentanones. On the contrary, the carbonyl absorption band is less prominent with Cat_A and Cat_B, corroborating the GC-MS data, where the latter two catalysts mixtures have majority yield of phenol derivatives (largely guaiacols). While aromatic C=C stretching vibrations at 1514 cm^−1^ can be observed in all four catalytic mixtures, C-H deformations in CH_2_ and CH_3_ group (1450–1460 cm^−1^) are more abundant in Cat_A and Cat_B. Finally, guaiacols ring breathing is shown at ~1270 cm^−1^ [6].

Phenolic content is evident due to absorption bands across 3640–3530 (OH stretch) and 1390–1310 cm^−1^ (OH bend) in all cases, however the indicators of specific functional groups are more valuable. The presence of methoxy groups for guaiacol compounds are indicated by bands at 2820 cm^−1^ (C-H stretch in CH_3_-O) and around 1275–1200 cm^−1^ (C-O bending for ethers) [25]. The abundance of methoxy and methyl groups in Cat_A and Cat_B as detected by GC-MS is also supported by the by the presence of larger absorption band at 750 cm^−1^ showcasing at least monosubstituted alkyl chains due to C-H bending of CH_3_ [X]. This a crucial peak to be observed to confirm functionalisation of aromatic compounds where mono-substitution is more likely to be observed.

Due to the large presence of pentose and hexose monosaccharides (listed as derived sugars), it may reasonably be expected that anhydride CO-O-CO bending would be observed at around 1050–1040 cm^−1^ by a strong peak, which is clearly prominent within all spectra. For Cat_C, this peak is substantially more prominent, which is as expected due to the highest derived sugars content of 5.3%.

**Figure 5:**
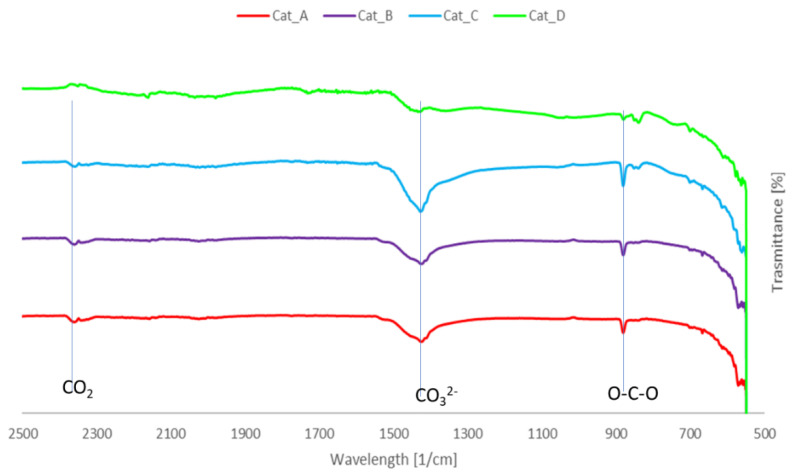
FTIR spectra of the spent catalytic mixtures.

The FTIR analysis of spent catalytic mixtures (Figure 5) seems to suggest condensed aromatics on the catalysts’ surface, as the absorption band at 1420 cm^−1^ is associated to aromatic skeletal vibration [6]. However, a deeper analysis and the congruent presence of adsorbed CO_2_ with band around 2360 cm^−1^ suggests that the broad band at 1427 cm^−1^ can be attributed to CO_3_^2-^ anion originating from sodium carbonate, as with the band at ~900 cm^−1^, due to deformation vibration of the atom in O-C-O bond of the same carbonate. This is suggestive of carbon dioxide adsorption under the studied conditions, which is confirmed by previous studies [26], and at the same time indicates that these materials could be of interest for processes such as CO_2_ sorption enhanced gasification. Moreover, additional analysis is required to confirm the presence of coke on the catalysts’ surface.

#### 2.2.3. Elemental Analysis

Elemental analysis was conducted on the catalytic mixtures before and after pyrolysis (see Table 4). Carbon content increase after pyrolysis is indicative of the coke build-up on the mixtures, with nitrogen content increasing. 

The change in the coked catalytic mixtures shows that, whilst there was a significant increase in carbon content due to coking on the raw mixtures, the per cent hydrogen content decreased, indicating the presence of saturated hydrocarbon components such as condensed aromatics low in H due to C=C bonds. The elemental analysis suggests that Cat_A and Cat_B are the most resilient to coke formation if compared to Cat_C, Cat_D and previously tested NaZrO_2_ (9.6 wt%) [18]. This is in agreement with previous works, where ceria has been linked to enhanced coke resistance and surface area by increasing porosity in CeNi-olivine catalyst [27] and ZrO_2_-CeO_2_ support improved thermal stability and resistance to coking [28].

## 3. Materials and Methods

### 3.1. Materials

The Etek lignin resulted from ethanol production by a two-stage weak acid hydrolysis of softwood with a lignin and holocellulose content, respectively, of 60 and 40 wt% [18]. Na_2_CO_3_ (99.6% purity, Sigma-Aldrich, Darmstadt, Germany), CeO_2_ (99.0% purity, Sigma-Aldrich) and ZrO_2_ (99.0% purity, Sigma-Aldrich) were used for the catalytic mixtures’ synthesis.

### 3.2. Catalytic Mixtures Synthesis

Metal oxide catalytic mixtures were prepared as such to contain varied ratios of the metal oxides of sodium, cerium and zirconium post calcination, as shown in Table 5. 

The samples were mixed using a pestle and mortar and then underwent calcination in an electric furnace to the parameters specified in Table 6. The defined temperature for calcination was 900 °C based on previous works that showed a transition from amorphous to crystalline ZrO_2_ at 800 °C, hence it was predicted that the synthesised catalytic mixtures would consist of various crystalline phases [14]. 

During calcination, Na_2_CO_3_ evolves carbon dioxide gas leading to the formation of sodium oxide, as shown in Equation (1), as this process is well understood. Na_2_ZrO_3_ may be present in crystalline form (2) or (3), as has been readily observed previously [13]. A key consideration must be the degree of formation of Na_2_O versus Na_2_ZrO_3_ or combinations thereof. ZrO_2_-CeO_2_ structures formed above calcination 540 °C have been studied, showing tetragonal and cubic crystalline phases [14].

(1) $$Na_{2}CO_{3\left(s\right)}\to Na_{2}O_{\left(s\right)}+CO_{2\left(g\right)}$$

(2) $$Na_{2}O_{\left(s\right)}+ZrO_{2}{}_{\left(s\right)}\to Na_{2}ZrO_{3}{}_{\left(s\right)}$$

(3) $$Na_{2}CO_{3\left(s\right)}+ZrO_{2\left(s\right)}\to Na_{2}ZrO_{3\left(s\right)}+CO_{2\left(g\right)}$$

The overall phases present may therefore be somewhat consistent with Equation (4). Phases present may indicate the dominant catalytic mixtures characteristics.

(4) $$wNa_{2}O+yZrO_{2}+xCeO_{2}\to Na_{w}Ce_{x}Zr_{y}O_{z}$$

### 3.3. Experimental and Calculations

**Figure 6:**
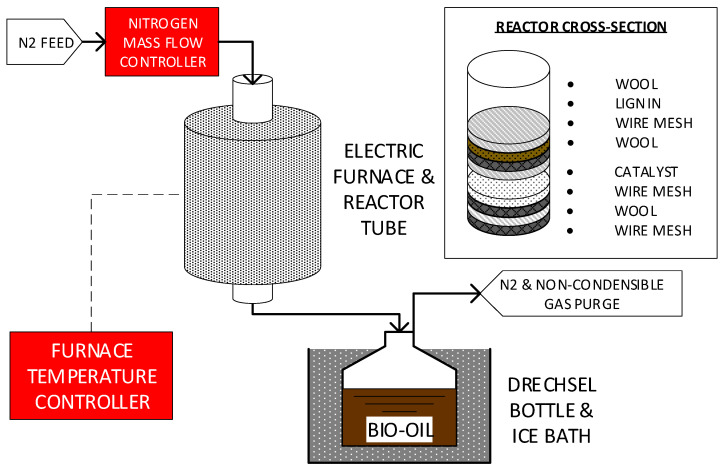
Experimental Apparatus.

The apparatus used was analogous to that used in comparable studies [18]. A 1" diameter 316 SS tube was used as the fixed bed reactor, with internal elements stacked as shown in Figure 6. Active components in the reactor are the lignin and catalyst (catalytic mixture), which were loaded at 1:1 mass ratio. An electric furnace with temperature controller was used to supply and regulate the heat for pyrolysis applying the parameters described in Table 6. 

Reaction vapour products were condensed using two 125 mL Dreschel bottles submerged in an ice-salt bath (−10 °C). A detailed description of the reactor set-up and method can be found in [18]. Mass of reactor and Dreschel bottles was taken prior to and post reaction to record the masses of bio-oil obtained. The condensed oil was removed from the Dreschel bottles using 20 mL acetone and the collected oil/acetone mixtures were left in fume cupboard overnight at ambient temperature to allow acetone evaporation. Then, the bio-oils were stored in fridge for further analysis. Hence, reaction conversion was taken on a mass basis as a percentage of original mass of lignin converted into pyrolysis products, as shown in Equation (5).

(5) $$X=\frac{\Delta m_{reactor}}{m_{Lignin}}$$

Subsequently, the bio-oil mass was measured directly, being taken as the mass increase in the Dreschel bottle (and steel tube fixture affixed to the reactor). Hence, the wt% of bio-oil when considering overall products (bio-oil, char and gas) was determined using Equation (6).

(6) $$W_{Oil}\left[wt\%\right]=\frac{m_{Oil}\left[g\right]}{m_{Lignin}\left[g\right]}\times 100\%$$

The mass of char was taken as the mass of lignin remaining within the reactor; hence, the wt% of char product was determined as shown in Equation (7).

(7) $$W_{Char}\left[wt\%\right]=\frac{\left(m_{Lignin}-\Delta m_{Reactor}\right)\left[g\right]}{m_{Lignin}\left[g\right]}\times 100\%$$

The mass of gaseous product was taken as the remaining mass different after considering wt% of all other products as the gas production rate was not measured post Dreschel bottles. Hence, the wt% of gas was determined as shown in Equation (8).

(8) $$W_{Gas}\left[wt\%\right]=100\%-\left(W_{Oil}+W_{Char}\right)=\left[wt\%\right]$$

### 3.4. Products Analysis

GC–MS analysis was performed using a Shimadzu GCMS QP2010 SE equipped with a Restek RXI-5HT column (30 m). A bio-oil sample of 1 μL (25% bio-oil–75% acetone) was injected at 290 °C under a pressure of 95.3 kPa. The oven was programmed to hold at 40 °C for 10 min, ramp at 5 °C/min to 200 °C and hold for 10 min, ramp at 10 °C/min to 250 °C and hold for 10 min, ramp at 10 °C/min to 300 °C and hold for 10 min. The data were collected by the integrated workstation software Shimadzu GCMSsolution, version 4.30 in duplicates to ensure reproducibility. Data were then analysed in Excel 2007 (version 12) environment, considering the compounds with a retention time of up to 45 min and an abundance ≥1% based on percentage area from GC-MS traces (see Tables S1–S4 in supplementary materials). The dataset was categorised by Principal Component Analysis (PCA) into functional groups: phenols (Ph), hydrocarbons (HC), Aldehydes (Alde), ketones (Ket), acids, derived sugars (DS), aromatics (AR), polyaromatics hydrocarbons (PAHs), alcohols (OH), cyclopentanones (CPO) and others. Fourier-transform infrared spectroscopy (FTIR) esd carried out using a Perkin Elmer Frontier assisted with Spectrum 10 software to acquire and process data. All absorption spectra were obtained in the 4000–500 cm^−1^ range. Good contact between sample and ATR-crystal surface was ensured before all measurements, while elemental analysis (EA) was done using an Exeter CE-440 Elemental analyser, Exeter Analytical (UK) Limited, UK. The tests were carried out in triplicates by weighting samples of 20 mg in microbalance and averages of C, H and N were considered. O was calculated by difference (O wt% = 100 − (C wt% + H wt% + N wt%). Therefore, O indicates other compounds present including metal oxides, oxygen in any potentially adsorbed CO_2(g)_, H_2_O_(l)_ and any O content from organic components.

## 4. Conclusions

In this study, mixed metal oxide catalytic mixtures derived from Na_2_CO_3_, CeO_2_ and ZrO_2_ were evaluated for the ex-situ catalytic pyrolysis of Etek lignin with the aim of decreasing coke formation related to pure Na_2_ZrO_3_ catalyst but retaining the capacity of the latter to produce phenols-rich bio-oil. Of the catalytic mixtures studied, equimolar metal oxides content (Cat_A) appears to have the optimal characteristics for renewable production of phenolic compounds with reduced coking and a recovery of 36 wt% of the products in bio-oil, which included significant methyl phenols (23.5%) and phenol (4.6%), suggesting demethoxylation activity of guaiacyl and syringyl monolignols. Moreover, Cat_A (together to Cat_B) more than halved the formation of coke (to 4 wt%) compared to previously tested pure NaZrO_2_ as well as Cat_C and Cat_D, in which the molar presence of ceria and zirconia were predominant. Predominance of ceria in the catalytic mixture (Cat_C) favoured the increase in conversion of the lignin waste holo-cellulose fraction, enriching the bio-oil in aldehydes, ketones and cyclopentanones, which increased the heterogeneity of the bio-oil and therefore its intrinsic quality in terms of phenols recovery. 

## Figures and Tables

**Table 1:** Products distribution.

				wt%		
Catalyst	Material	Conversion	Oil	Char	Gases	Source
Cat_A	Etek Lignin	71	36	29	35	
Cat_B	Etek Lignin	73	33	27	40	
Cat_C	Etek Lignin	72	46	28	26	
Cat_D	Etek Lignin	73	30	27	43	
None	Etek Lignin	73 *	58	27	10	[20]
Na:Zr (1.5:1)	Etek Lignin	72	41	28	31	[18]

* Calculated from source data using the methods applied in this study.

**Table 2:** Bio-oil product functional group distribution obtained from GC-MS analysis.

					Aarea%						
Catalyst	Ph	HC	Alde	Ket	Acids	DS	AR	PAHs	OH	CPO	Others
Cat_A	55.5	8.4	3.8	7.2	2.0	2.1	3.9	2.9	6.7	5.6	1.9
Cat_B	67.7	3.8	1.1	4.2	2.6	1.3	2.3	6.7	4.1	5.9	0.2
Cat_C	41.2	6.3	7.4	11.8	1.2	5.2	2.0	4.2	4.6	15.9	0.3
Cat_D	52.9	5.3	3.9	4.8	5.3	1.4	6.3	5.2	3.2	9.5	2.2
None [20]	48.3	0.0	14.3	12.0	3.0	15.0	3.5	0.0	4.0	0.0	0.0

Phenols (Ph), hydrocarbons (HC), Aldehydes (Alde), Ketones (Ket), Acids, Derived Sugars (DS), Aromatics (AR), Polyaromatics Hydrocarbons (PAHs), Alcohols (OH), Cyclopentanones (CPO).

**Table 3:** Phenol distribution of bio-oil products obtained from GC-MS analysis.

					Area%			
Catalyst	Pyrolysis Material	Phenol	Guaiacols	Methyl Phenols	Catechols	Alkyl Phenols	Others	Source
Cat_A	Etek lignin	4.6	58.9	23.5	6.5	4.6	1.9	
Cat_B	Etek lignin	4.6	64.8	18.5	2.5	4.2	5.4	
Cat_C	Etek lignin	3.2	59.3	15.9	11.7	8.3	1.6	
Cat_D	Etek lignin	4.0	67.3	18.2	4.4	5.2	0.9	
None	Etek lignin	1.1	89.0	6.5	3.3	0.1	0.0	[20]

**Table 4:** Elemental analysis of the raw and spent catalytic mixtures and wt% change in coke (coke _spent cat_−coke _raw cat_).

	wt%, Dry Basis				wt%, Dry Basis				wt%, Dry Basis		
Catalytic Mixture	Raw Catalytic Mixture Data				Spent (Coked) Catalytic Mixture Data				Change in Coked Catalytic Mixture		
	C	H	N	O	C	H	N	O	C	H	N *
Cat_A	1.68	0.46	-	97.86	5.60	0.18	0.01	94.21	3.92	−0.28	0.01
Cat_B	2.59	0.63	-	96.78	6.87	0.31	0.05	92.77	4.28	−0.32	0.05
Cat_C	0.49	0.07	-	99.44	9.08	0.24	0.05	90.63	8.59	0.17	0.05
Cat_D	1.80	0.37	-	97.83	10.12	0.44	0.02	89.42	8.32	0.07	0.02

* Difference between spent and raw catalytic mixtures, hence the value presented shows the major difference post.

**Table 5:** Catalytic mixtures’ molar composition post calcination and their predicted mass fraction.

Catalytic Mixtures	Molar Ratio of Compound			Predicted Mass Fraction of Calcined Mixtures Components, wt%			
	Na_2_O	CeO_2_	ZrO_2_	Na_2_O	CeO_2_	ZrO_2_	Total
Cat_A	1	1	1	17.4	48.2	34.5	100.0
Cat_B	2	1	1	29.6	41.0	29.4	100.0
Cat_C	1	2	1	11.7	65.0	23.3	100.0
Cat_D	1	1	2	12.9	35.8	51.3	100.0

**Table 6:** Experimental Parameters.

Parameter	Calcination	Pyrolysis
Hold-Temperature [°C]	900	600
Ramp Rate [°C]	5	100
Hold-Time	2 h	15 min
N_2_ Flowrate [mL/min]	-	100

## Data Availability

The data presented in this study are available on request from the corresponding authors

## Supplementary Materials

The following are available online. Table S1. GC-MS data for Cat_A; Table S2. GC-MS data for Cat_B; Table S3. GC-MS data for Cat_C; Table S4. GC-MS data for Cat_D.

## References

[1] Anastas P.T., Warner J.C. (1998). Green Chemistry: Theory and Practice.

[2] Plotkin J.S. (2016). What’s New in Phenol Production. Am. Chem. Soc..

[3] Jiang G., Nowakowski D.J., Bridgwater A.V. (2009). A systematic study of the kinetics of lignin pyrolysis. Thermochem. Acta.

[4] Sun Y., Cheng J. (2002). Hydrolysis of lignocellulosic materials for ethanol production: A review. Bioresour. Technol..

[5] Sekab (2019). Biorefinery Technology. https://www.sekab.com/en/products-services/biorefinery/.

[6] Trubetskaya A., Lange H., Wittgens B., Brunsvik A., Crestini C., Rova U., Christakopoulos P., Leahy J.J., Matsakas L. (2020). Structural and Thermal Characterization of Novel Organosolv Lignins from Wood and Herbaceous Sources. Processes.

[7] Magalhães D., Gürel K., Matsakas L., Christakopoulos P., Pisano I., Leahy J.J., Kazanç F., Trubetskaya A. (2021). Prediction of yields and composition of char from fast pyrolysis of commercial lignocellulosic materials, organosolv fractionated and torrefied olive stones. Fuel.

[8] Sanna A., Vispute T.P., Huber G.W. (2015). Hydrodeoxygenation of the aqueous fraction of bio-oil with Ru/C and Pt/C catalysts. Appl. Catal. B Environ..

[9] Stummann M.Z., Høj M., Hansen A.B., Wiwel P., Davidsen B., Jensen P.A., Jensen A.D. Hydrogen assisted catalytic biomass pyrolysis for green fuels. Proceedings of the Effect of Catalyst in the Fluid Bed. Abstract from The 10th International Conference on Environmental Catalysis, The 3rd International Symposium on Catalytic Science and Technology in Sustainable Energy and Environment.

[10] IH2® Technology, Shell. https://www.shell.com/business-customers/catalysts-technologies/licensed-technologies/benefits-of-biofuels/ih2-technology.html.

[11] Zhang S., Yang X., Zhang H., Chu C., Zheng K., Ju M., Liu L. (2019). Liquefaction of Biomass and Upgrading of Bio-Oil: A Review. Molecules.

[12] Jeon M.-J., Jeon J.-K., Suh D.S., Park S.H., Sa J.Y., Joo S.H., Park Y.-K. (2013). Catalytic pyrolysis of biomass components over mesoporous catalysts using Py-GC/MS. Catal. Today.

[13] Behrens M., Cross J.S., Akasaka H., Ohtake N. (2017). A study of guaiacol, cellulose and Hinoki wood pyrolysis with silica, ZrO_2_ & TiO_2_ and ZSM-5 catalysts. J. Anal. Appl. Pyrolysis.

[14] Grams J., Niewiadomski M., Ruppert A.M., Kwapinski W. (2015). Catalytic performance of a Ni Catalyst Supported on CeO_2_, ZrO_2_ and CeO_2_-ZrO_2_ in the upgrading of cellulose fast pyrolysis vapours. Competes Rendus Chim..

[15] Kumar A., Biswas B., Bhaskar T. (2020). Effect of cobalt on titania, ceria and zirconia oxide supported catalysts on the oxidative depolymerization of prot and alkali lignin. Bior. Technol..

[16] Maki-Arvela P., Murzin D.Y. (2017). Hydrodeoxygenation of Lignin-Derived Phenols: From Fundamental Studies towards Industrial Applications. Catalysts.

[17] Memon M.Z., Ji G., Li J., Zhao M. (2017). Na_2_ZrO_3_ as an Effective Bifunctional Catalyst-Sorbent during Cellulose Pyrolysis. I&EC Res..

[18] Hendry A., Åhlén M., Fernandes T., Cheung O., Sanna A. (2020). Catalytic cracking of Etek lignin with zirconia supported metal-oxides for alkyl and alkoxy phenols recovery. Bioresour. Technol..

[19] Wan S., Wang Y. (2014). A review on ex situ catalytic fast pyrolysis of biomass. Front. Chem. Sci. Eng..

[20] Nowakowski D.J., Bridgwater A.V., Elliott D.C., Meier D., de Wild P. (2009). Lignin fast pyrolysis: Result from an international collaboration. J. Anal. Appl. Pyrolysis.

[21] Lu Q., Zhang Y., Tang Z., Li W.-Z., Zhu X.-F. (2010). Catalytic upgrading of biomass fast pyrolysis vapors with titania and zirconia/titania based catalysts. Fuel.

[22] Mante O.D., Rodriguez J.A., Senanayake S.D., Babu S.P. (2015). Catalytic conversion of biomass pyrolysis vapors into hydrocarbon fuel precursors. Green Chem..

[23] (2020). Merck kGaA. IR Spectrum Table & Chart. https://www.sigmaaldrich.com/technical-documents/articles/biology/ir-spectrum-table.html.

[24] Zhang H., Shao S., Xiao R., Shen D., Zeng J. (2014). Characterization of Coke Deposition in the Catalytic Fast Pyrolysis of Biomass Derivates. Energy Fuels.

[25] Coates J., Meyers R.A. (2000). Interpretation of Infrared Spectra, a Practical Approach. Encyclopedia of Analytical Chemistry.

[26] Munro S., Åhlén M., Cheung O., Sanna A. (2020). Tuning Na_2_ZrO_3_ for fast and stable CO_2_ adsorption by solid state synthesis. Chem. Eng. J..

[27] Yang Y., Du Z., Huang Y., Lu F., Wang F., Gao J., Xu J. (2013). Conversion of furfural into cyclopentanone over Ni–Cu bimetallic catalysts. Green Chem..

[28] Ardiyanti A.R., Khromova S.A., Venderbosch R.H., Yakovlev V.A., Milian-Cabrera I.V., Heeres H.J. (2012). Catalytic hydrotreatment of fast pyrolysis oil using bimetallic Ni-Cu catalysts on various supports. Appl. Catal. A Gen..

