# Supplementary material for: Effect of Ceria Addition to Na2O-ZrO2 Catalytic Mixtures on Lignin Waste Ex-Situ Pyrolysis
Source: Molecules. 2021 Feb 5;26(4):827. doi: 10.3390/molecules26040827 (PMC7915913; doi:10.3390/molecules26040827)
Supplement: Supplementary file 1 [file molecules-26-00827-s001.pdf]

molecules

Article

Effect of Ceria Addition to Na_2O-ZrO_2 Catalytic Mixtures on Lignin Waste Ex-situ Pyrolysis

Adam Yeardley [1], Giuseppe Bagnato [2] and Aimaro Sanna [1,*]

[1] Advanced Biofuels Lab, Institute of Mechanical, Process and Energy Engineering, School of Engineering and Physical Sciences, Heriot-Watt University, Edinburgh EH14 4AS, UK; AJY1@hw.ac.uk
[2] School of Chemistry and Chemical Engineering, Queen's University Belfast, David Keir Building, Belfast BT9 5AG, UK; G.Bagnato@qub.ac.uk
* Correspondence: A.Sanna@hw.ac.uk

Appendix A. GC-MS Analysis

GC-MS data of the catalytic mixtures are summarized in Tables S1–S4.

Table S1. GC-MS data for Cat_A.

Peak#	Ret.Time	Area	Height	A/H	Conc.	Name
1	1.806	174563	130109	1.34	0.07	Propanal, 2-methyl-
2	2.251	171831	85517	2.01	0.07	Acetic acid
3	3.567	79106	33891	2.33	0.03	Propanoic acid
4	6.925	117638	42202	2.79	0.05	2-Cyclopenten-1-one
5	7.008	93138	29999	3.1	0.04	1,2,4-Triazole, 4-[*N*-(2-hydroxyethyl)-*N*-nitro]amino-
6	7.136	268085	142344	1.88	0.11	2-Pentanone, 4-hydroxy-4-methyl-
7	7.165	153077	122081	1.25	0.06	2-*t*-Butyl-4-methyl-5-oxo-[1,3]dioxolane-4-carboxylic acid
8	7.205	172572	86719	1.99	0.07	Propanedioic acid, mono(1,1-dimethylethyl) ester
9	7.235	166295	73530	2.26	0.07	Hydroperoxide, 1-ethylbutyl
10	14.348	106852	56236	1.9	0.04	2-Furancarboxaldehyde, 5-methyl-
11	16.272	5074778	1393125	3.64	2.1	Phenol
12	17.342	122860	26348	4.66	0.05	2-Cyclopenten-1-one, 3,4-dimethyl-
13	17.662	745658	498325	1.5	0.31	1,2-Cyclopentanedione, 3-methyl-
14	17.697	4427027	865897	5.11	1.83	2-Cyclopenten-1-one, 2,3-dimethyl-
15	18.241	167730	53822	3.12	0.07	3-Buten-1-ol, 3-methyl-2-methylene-
16	18.769	2235393	577269	3.87	0.92	2-Cyclopenten-1-one, 3-ethyl-2-hydroxy-
17	18.866	4310949	1640823	2.63	1.78	Phenol, 2-methyl-
18	18.962	199039	93234	2.13	0.08	3-Oxatricyclo[4.2.0.0(2,4)]octan-7-one
19	19.073	281369	57426	4.9	0.12	Bicyclo[5.2.0]nonane, 1,7-dimethyl-, cis-
20	19.146	505293	165059	3.06	0.21	2-Cyclopenten-1-one, 3-ethyl-
21	19.217	209946	94763	2.22	0.09	6-Hydroxyhexahydrocyclopenta[b]furan-2-one
22	19.268	88930	42791	2.08	0.04	1-(5,5,5-Trichloropentyl)-1*H*-1,2,4-triazole
23	19.335	12663205	3763151	3.37	5.23	Phenol, 2-methoxy-
24	19.715	8351198	1859822	4.49	3.45	Phenol, 3-methyl-
25	20.102	132992	51448	2.58	0.05	3-Methylene-2-norbornanone
26	20.173	699616	170640	4.1	0.29	Phenol, 2,6-dimethyl-
27	20.912	757631	168944	4.48	0.31	2-Cyclopenten-1-one, 3-ethyl-2-hydroxy-
28	21.022	432135	148848	2.9	0.18	Cyclohexanone, 3-ethenyl-
29	21.098	220926	66080	3.34	0.09	Creosol
30	21.412	91859	42621	2.16	0.04	Cyclohexane, 1-propenyl-
31	21.488	379508	81544	4.65	0.16	Benzene, 1,2-dimethoxy-

32	21.683	971232	305342	3.18	0.4	4-Isopropylidene-cyclohexanol
33	21.804	757240	223998	3.38	0.31	Bicyclo[2.2.2]octan-1-ol, 4-methyl-
34	21.899	2481553	1107078	2.24	1.02	Phenol, 2,4-dimethyl-
35	21.936	2030320	750258	2.71	0.84	Phenol, 2,6-dimethyl-
36	22.055	94494	33278	2.84	0.04	4*H*-1,2,4-Triazole-3-thiol, 4-(2-fluorophenyl)-5-(1-methylethyl)-
37	22.121	251210	52405	4.79	0.1	Cyclohexene, 1-methoxy-
38	22.218	130491	34276	3.81	0.05	Azulene
39	22.288	120254	56211	2.14	0.05	1-Methylcyclooctene
40	22.346	1153583	456401	2.53	0.48	Creosol
41	22.626	3547330	1325742	2.68	1.46	Phenol, 2,3-dimethyl-
42	22.686	903471	322522	2.8	0.37	Phenol, 3-ethyl-
43	22.8	21346135	7974195	2.68	8.81	Creosol
44	23.258	473519	142319	3.33	0.2	Phenol, 2,3,5-trimethyl-
45	23.363	1598396	579093	2.76	0.66	Phenol, 3,4-dimethyl-
46	23.618	226907	99012	2.29	0.09	2-Hydroxy-3-propyl-2-cyclopenten-1-one
47	23.691	2429303	777356	3.13	1	1,4:3,6-Dianhydro-.alpha.-d-glucopyranose
48	23.768	1457389	445646	3.27	0.6	Heptanal
49	23.905	142707	33877	4.21	0.06	Butanoic acid, 2-methyl-, 3,7-dimethyl-6-octenyl ester
50	23.994	687373	218930	3.14	0.28	2,3-Anhydro-d-mannosan
51	24.058	493534	162691	3.03	0.2	Phenol, 2,4,6-trimethyl-
52	24.245	3179015	540887	5.88	1.31	Catechol
53	24.38	477572	145222	3.29	0.2	Phenol, 2,3,6-trimethyl-
54	24.444	402044	129047	3.12	0.17	2,3-Anhydro-d-mannosan
55	24.565	1655312	423982	3.9	0.68	Phenol, 3,4,5-trimethyl-
56	24.665	1002592	274792	3.65	0.41	Phenol, 2-ethyl-6-methyl-
57	24.785	161948	76110	2.13	0.07	*p*-Acetylphenyl-.beta.-D-glucoside
58	24.84	433714	140915	3.08	0.18	2,3-Dimethoxytoluene
59	24.872	177855	101159	1.76	0.07	2,3-Anhydro-d-mannosan
60	24.892	290462	102345	2.84	0.12	1*H*-Azepin-1-amine, hexahydro-
61	25.236	799119	318511	2.51	0.33	Phenol, 2-ethyl-5-methyl-
62	25.331	10350279	3344317	3.09	4.27	Phenol, 4-ethyl-2-methoxy-
63	25.475	904826	290875	3.11	0.37	Phenol, 2,3,6-trimethyl-
64	25.661	83216	33044	2.52	0.03	(1-Ethylpyrrolidin-3-yl)methanamine
65	25.759	2171916	521168	4.17	0.9	1,2-Benzenediol, 4-methyl-
66	25.907	353456	83735	4.22	0.15	1-[3-Hydroxy-4-(3,4,5-trihydroxy-6-hydroxymethyltetrahydropyran-2-yloxy)phenyl]ethanone
67	25.978	236234	96091	2.46	0.1	Naphthalene, 1-methyl-
68	26.038	477560	128304	3.72	0.2	1H-Indene-1,2-diol, 2,3-dihydro-1-methyl-, cis-
69	26.165	347738	56070	6.2	0.14	Benzenamine, N,N,3-trimethyl-
70	26.293	489827	220537	2.22	0.2	3′,5′-Dihydroxyacetophenone
71	26.346	2956098	1036210	2.85	1.22	2-Methoxy-4-vinylphenol
72	26.428	494537	153827	3.21	0.2	4,6-Decadiyne
73	26.538	241849	63017	3.84	0.1	Phenol, 4-(2-propenyl)-
74	26.629	1875942	465172	4.03	0.77	1,2-Benzenediol, 4-methyl-
75	26.767	1526921	314179	4.86	0.63	1,4-Benzenedimethanol, .alpha.-methyl-
76	26.845	167701	117573	1.43	0.07	2-Propen-1-ol, 3-(2,6,6-trimethyl-1-cyclohexen-1-yl)-
77	26.872	162324	108740	1.49	0.07	Estr-4-en-3-one
78	26.938	251784	87699	2.87	0.1	Benzimidazole, 2-methoxy-, 3-oxide
79	26.985	214686	105620	2.03	0.09	2-Methyl-4-propylphenol

80	27.038	766577	287854	2.66	0.32	3-Methoxy-5-methylphenol
81	27.092	186263	82915	2.25	0.08	Methoxy-phenyl-acetic acid (5-nitro-thiophen-2-ylmethylene)-hydrazide
82	27.209	209194	57349	3.65	0.09	7-Methylindan-1-one
83	27.272	232192	94803	2.45	0.1	Benzofuran, 7-methyl-
84	27.335	388516	100502	3.87	0.16	Cyclohexene, 4-(2-(ethoxycarbonyl)ethenyl)-5-methyl-
85	27.426	773427	270096	2.86	0.32	Phenol, 3-methoxy-2,4,5-trimethyl-
86	27.487	2358382	907002	2.6	0.97	Phenol, 2-methoxy-3-(2-propenyl)-
87	27.565	1361458	411908	3.31	0.56	trans-4-Methoxycinnamaldehyde
88	27.724	2631053	880671	2.99	1.09	Phenol, 2-methoxy-4-propyl-
89	27.882	729485	147498	4.95	0.3	Phenol, 2,3,5,6-tetramethyl-
90	28.045	782266	277145	2.82	0.32	p-Octyloxybenzyl alcohol
91	28.092	1535523	372570	4.12	0.63	1,3-Benzenediol, 2,5-dimethyl-
92	28.233	143965	78483	1.83	0.06	cis-p-Mentha-2,8-dien-1-ol
93	28.258	229004	85024	2.69	0.09	1-Cyclohexene-1-acetaldehyde, 2,6,6-trimethyl-
94	28.325	367206	93489	3.93	0.15	Naphth[1,2-b]oxirene, 1a,2,3,7b-tetrahydro-
95	28.439	205030	56031	3.66	0.08	5-(4-Methoxybenzylidene)-3-(p-tolyl)rhodanine
96	28.528	370278	102586	3.61	0.15	Phenol, 2-methoxy-5-(1-propenyl)-, (E)-
97	28.693	3129246	911438	3.43	1.29	Vanillin
98	28.822	1868097	672984	2.78	0.77	Phenol, 2-methoxy-4-(1-propenyl)-
99	28.892	288090	109044	2.64	0.12	2,3-Dimethylhydroquinone
100	28.944	267178	104206	2.56	0.11	Bromotrimethylsilane
101	29.018	275085	137801	2	0.11	3-Buten-2-one, 4-(2,2,6-trimethyl-7-oxabicyclo[4.1.0]hept-1-yl)-
102	29.032	369155	136827	2.7	0.15	Silane, bromomethyltripropyl-
103	29.145	184772	72555	2.55	0.08	2-Methoxyphenylacetone
104	29.206	425075	121284	3.5	0.18	Benzenamine, N,N-diethyl-4-nitroso-
105	29.291	659104	186020	3.54	0.27	Phenol, 5-methoxy-2,3-dimethyl-
106	29.348	268848	120093	2.24	0.11	2-Allyl-4-methylphenol
107	29.405	156789	59673	2.63	0.06	m-Phenylenediamine, TMS derivative
108	29.502	109058	71645	1.52	0.05	Phosphine, (pentamethylphenyl)-
109	29.543	525887	120093	4.38	0.22	Coniferyl aldehyde
110	29.645	243521	92736	2.63	0.1	Benzaldehyde, 4-(1-methylethyl)-
111	29.705	568339	183383	3.1	0.23	Benzo[b]thiophene 1,1-dioxide, 3-methyl-
112	29.846	6777773	2605812	2.6	2.8	Phenol, 2-methoxy-4-(1-propenyl)-, (Z)-
113	29.962	533506	86863	6.14	0.22	2-(Benzooxazol-2-ylsulfanyl)-N-furan-2-ylmethyl-acetamide
114	30.028	187756	115296	1.63	0.08	Octahydroindene-1,7a-diol
115	30.077	322860	123251	2.62	0.13	6-Methyl-4-indanol
116	30.155	205375	65899	3.12	0.08	Cyclobutene, 1-cyclopropyl-2-ethoxy-3,3-difluoro-
117	30.211	428498	147983	2.9	0.18	1H-Indene, 5,6-dimethoxy-
118	30.28	464436	135833	3.42	0.19	Phenol, 2-methoxy-3-(2-propenyl)-
119	30.332	170993	114106	1.5	0.07	Phenol, 3,5-dimethyl-2-nitro-
120	30.355	155469	119615	1.3	0.06	2-Acetyl-5,6,7,8-tetrahydroquinoxaline
121	30.408	555530	170484	3.26	0.23	1-Naphthalenol, 5,8-dihydro-
122	30.438	315971	141335	2.24	0.13	Benzene, (trifluoromethyl)-
123	30.513	541196	153472	3.53	0.22	2-Ethyl-4-chromanol, (cis)-
124	30.615	87278	34841	2.51	0.04	Cyclohexane, (3-chloro-1-propynyl)-
125	30.68	203431	86935	2.34	0.08	Benzaldehyde, 2,5-dimethoxy-
126	30.732	277046	84413	3.28	0.11	Tricyclo[3.2.1.02,7]oct-3-ene, 2,3,4,5-tetramethyl-
127	30.865	1881359	569607	3.3	0.78	Apocynin

128	30.995	274850	86622	3.17	0.11	Benzene, 1,2-dimethoxy-4-propenyl-, (*Z*)-
129	31.062	132330	55905	2.37	0.05	Benzeneethanol, .alpha.-methyl-3-(1-methylethyl)-
130	31.114	310908	86581	3.59	0.13	dl-3Beta-hydroxy-d-homo-18-nor-5alpha,8alpha,14beta-androst-13(17a)-en-17-one
131	31.229	304677	77406	3.94	0.13	p-Ethoxybenzyl alcohol
132	31.335	612478	188169	3.25	0.25	Benzenemethanol, 3,4-dimethoxy-
133	31.381	761928	260837	2.92	0.31	1,8-Naphthalenedione, 8a-ethylperhydro
134	31.468	1438814	317195	4.54	0.59	3,6,9-Trioxa-12-azadocosan-1-ol, TMS derivative
135	31.528	948211	316836	2.99	0.39	2-Methyl-l-methylmannopyranoside
136	31.575	595486	357358	1.67	0.25	4-Hydroxy-1,3-dimethyl-piperidine-4-carbonitrile
137	31.628	2372528	434601	5.46	0.98	Benzoic acid, 4-hydroxy-3-methoxy-, methyl ester
138	31.705	748541	377374	1.98	0.31	1-Silacyclopenta-2,4-diene, 3-ethyl-1,1,2,5-tetramethyl-
139	31.718	529286	388471	1.36	0.22	Alpha-l-rhamnopyranose
140	31.756	717823	417209	1.72	0.3	.beta.-D-Glucopyranose, 1,6-anhydro-
141	31.785	603237	471993	1.28	0.25	[1,3]Dithiolan-2-ylidene-fluoro-acetonitrile
142	31.806	1129727	490632	2.3	0.47	Sedoheptulosan
143	31.882	5450518	1107043	4.92	2.25	2-Propanone, 1-(4-hydroxy-3-methoxyphenyl)-
144	31.982	1276776	604698	2.11	0.53	Cinnamic acid, 4-hydroxy-3-methoxy-, (5-hydroxy-2-hydroxymethyl-6-[2-(4-hydroxy-3-methoxyphenyl)ethoxy]-4-(6-methyl-3,4,5-trihy
145	32.005	812316	584199	1.39	0.34	Chloroacetic acid, 1-naphthyl ester
146	32.022	218749	566682	0.39	0.09	2-Thioxo-imidazolidin-4-one-5-propanolic acid
147	32.032	896297	575213	1.56	0.37	1,2,3,4-Cyclopentanetetrol, (1.alpha.,2.beta.,3.beta.,4.alpha.)-
148	32.058	335371	563741	0.59	0.14	*t*-Butyl 1-thio-.alpha.-D-glucopyranoside
149	32.102	1644585	635013	2.59	0.68	3,4-Altrosan
150	32.115	876378	655803	1.34	0.36	1-(p-Toluidino)-1-deoxy-.beta.-d-idopyranose
151	32.172	5213889	758097	6.88	2.15	2,3,5,6-Tetramethylbenzoic acid
152	32.278	2963983	503089	5.89	1.22	Carbamic acid, (1-methylethyl)-, 2-[[(aminocarbonyl)oxy]methyl]-2-methylpentyl ester
153	32.432	430860	220206	1.96	0.18	Pyrano[4,3-b]benzopyran-1,9-dione,5a-methoxy-9a-methyl-3-(1-propenyl)perhydro-
154	32.455	245600	210006	1.17	0.1	Glutaric acid, hept-2-yl geranyl ester
155	32.479	540108	220566	2.45	0.22	Polygalitol
156	32.508	667440	204141	3.27	0.28	.beta.-D-Glucopyranoside, 4-nitrophenyl
157	32.652	451598	120922	3.73	0.19	2-Octenoic acid
158	32.692	328450	113769	2.89	0.14	o-Cresol, TMS derivative
159	32.731	305894	105323	2.9	0.13	3-Buten-2-one, 4-(2-methoxyphenyl)-
160	32.832	696278	137123	5.08	0.29	1*H*-Inden-1-one, 2,3-dihydro-3,3-dimethyl-
161	32.918	124155	54664	2.27	0.05	Ethyl 9,9-diformylnona-2,4,6,8-tetraenoate
162	32.968	129588	48596	2.67	0.05	Benzenesulfonamide, 4-butyl-*N*-(5-methylisoxazol-3-yl)-
163	33.078	991114	259690	3.82	0.41	1-(2-Hydroxy-4-methoxyphenyl)propan-1-one
164	33.242	799158	164782	4.85	0.33	Diethyl Phthalate
165	33.335	166653	51817	3.22	0.07	3-n-Butylthiane,S-oxide
166	33.405	151089	42228	3.58	0.06	1,2,4-Trioxolane-2-octanoic acid, 5-octyl-, methyl ester
167	33.632	83318	23365	3.57	0.03	3-(Prop-2-en-1-onyl)-2,4,4-trimethylcyclohex-2-en-1-one
168	33.711	98389	46110	2.13	0.04	*N*-Methylbenzylamine, TMS derivative
169	33.909	107948	37277	2.9	0.04	1*H*-Purin-2-amine, 6-methoxy-*N*-methyl-
170	34.038	325261	94636	3.44	0.13	Benzene, 4-methyl-1,2-dinitro-
171	34.075	177376	68398	2.59	0.07	2-Cyclopenten-1-one, 3-phenyl-

172	34.122	123734	43979	2.81	0.05	Imidazo[3,4-c]pyridin-6(3*H*)-on-1-propanoic acid, 4,5,6,7-tetrahydro-5-benzyl-, ethyl ester
173	34.289	736835	135072	5.46	0.3	5-Nitro-2,3,6-trimethylindole
174	34.352	125660	101222	1.24	0.05	Coniferyl aldehyde, 2-methylpropyl ether
175	34.372	251516	86045	2.92	0.1	Propionamide, 2-(2,4-dichlorophenoxy)-*N*-(1-ethyl-1-methylprop-2-ynyl)-
176	34.472	217364	77493	2.8	0.09	2-Butanone, 4-(4-hydroxy-3-methoxyphenyl)-
177	34.545	139437	54292	2.57	0.06	Allenyl o-nitrophenyl sulfide
178	34.58	191505	71964	2.66	0.08	4-Methyl-6-methoxycoumarin
179	34.684	1734982	342661	5.06	0.72	Benzenepropanol, 4-hydroxy-3-methoxy-
180	34.814	462053	155069	2.98	0.19	2-Naphthalenol, 3-methoxy-
181	35.173	159174	67354	2.36	0.07	3*H*-3a,7-Methanoazulene, 2,4,5,6,7,8-hexahydro-1,4,9,9-tetramethyl-, [3aR-(3a.alpha.,4.beta.,7.alpha.)]-
182	35.377	138377	55077	2.51	0.06	Benzene, 1,2,3,4-tetramethyl-4-(1-methylethenyl)-
183	35.511	218251	56233	3.88	0.09	Phenylacetylformic acid, 4-hydroxy-3-methoxy-
184	36.208	236302	68434	3.45	0.1	Hexa-t-butylcyclotrisilane
185	36.295	198138	63667	3.11	0.08	.beta.-Alanine, *N*-(2-chloroethoxycarbonyl)-, isohexyl ester
186	36.594	104053	40986	2.54	0.04	Naphthalene, 1,2-dihydro-3,5,8-trimethyl-
187	36.694	209425	67933	3.08	0.09	1-(4-Tolyl)-1-cyclohexene
188	36.785	82125	33808	2.43	0.03	Isolongifolene-5-ol
189	37.124	475107	145244	3.27	0.2	Diphenylacetylene
190	37.338	88767	69740	1.27	0.04	2-(4-Chlorophenoxy)-N′-(2-propoxybenzylidene)acethydrazide
191	37.712	149479	69234	2.16	0.06	4-Hydroxy-3-methoxybenzyl alcohol, di(isopropyl) ether
192	38.095	180015	68565	2.63	0.07	.alpha.-Dehydro-ar-himachalene
193	38.361	129542	40178	3.22	0.05	4-(2,2,6-Trimethyl-7-oxabicyclo[4.1.0]hept-4-en-1-yl)pent-3-en-2-one
194	38.736	299084	69164	4.32	0.12	2-Hydroxy-4-isopropylnaphthalene
195	38.838	194829	66617	2.92	0.08	1,2-Benzenedicarboxylic acid, bis(2-methylpropyl) ester
196	39.052	154544	66227	2.33	0.06	Octanoic acid, 2,2-dichloro-3-hydroxy-, ethyl ester
197	39.075	201756	82458	2.45	0.08	Octadecanoic acid, 10-oxo-, methyl ester
198	39.178	130770	39650	3.3	0.05	5-Acetoxy-14-methyloxacyclotetradec-10-en-2-one
199	39.802	227424	80721	2.82	0.09	Hexadecanoic acid, methyl ester
200	40.466	97427	32532	2.99	0.04	5,10-Diethoxy-2,3,7,8-tetrahydro-1H,6H-dipyrrolo[1,2-a:1′,2′-d]pyrazine
201	40.541	85088	42829	1.99	0.04	Pyridazin-3(2H)-one, 4-isopropoxy-5-mercapto-2-phenyl-
202	40.638	187925	77167	2.44	0.08	Phthalic acid, butyl oct-3-yl ester
203	40.935	99578	16156	6.16	0.04	Cyclopropane, 1,1-dimethyl-2-phenylethynyl-2-propyl-
204	41.058	147200	35338	4.17	0.06	Hexanoic acid, 2-ethyl-2-propyl-, methyl ester
205	41.138	141567	34342	4.12	0.06	*N*-(4-Fluorophenyl)oxan-4-amine
206	41.66	109118	35237	3.1	0.05	1,1′-(4-Methyl-1,3-phenylene)bis[3-(5-isopropyl-1,3,4-thiadiazol-2-yl)urea]
207	41.76	137852	43224	3.19	0.06	10,12,14-Nonacosatriynoic acid
208	42.269	293041	87468	3.35	0.12	Phenanthrene, 2,5-dimethyl-
209	42.471	78392	27169	2.89	0.03	Spiro[benzofuran-3(2H),1′-[2,5]cyclohexadiene]-5-carboxylic acid, 6-hydroxy-2′-methoxy-2,4′-dioxo-4-pentyl-, methyl ester
210	43.177	108670	40776	2.67	0.04	10,18-Bisnorabieta-5,7,9(10),11,13-pentaene
211	43.705	96556	33220	2.91	0.04	4-Pyridinol, 3-chloro-2,6-dimethyl-5-nitro-
212	44.195	123374	29208	4.22	0.05	3-Acetylphenoxathiin
213	44.477	229207	78865	2.91	0.09	9-Ethyl-10-methylanthracene

214	44.907	127772	45904	2.78	0.05	Trichloroacetic acid, hexadecyl ester
215	45.093	80566	24579	3.28	0.03	1,3,2-diazaphosphorine, 1,3-diethylhexahydro-2-phenyl-, 2-oxide
216	45.644	756548	271934	2.78	0.31	Retene

Table S2. GC-MS data for Cat_B.

Peak#	Ret.Time	Area	Height	A/H	Conc.	Name
1	1.804	92416	106658	0.87	0.04	Propanal, 2-methyl-
2	1.862	31081	45730	0.68	0.01	Furan, 2,5-dihydro-
3	2.209	541803	302941	1.79	0.26	Acetic acid
4	2.577	135343	105206	1.29	0.06	2-Propanone, 1-hydroxy-
5	7.025	480850	143624	3.35	0.23	2-Pentanone, 4-hydroxy-4-methyl-
6	14.118	120165	39388	3.05	0.06	2*H*-Pyran-2-one, tetrahydro-
7	14.224	434192	112386	3.86	0.21	2-Furancarboxaldehyde, 5-methyl-
8	14.58	394494	114552	3.44	0.19	2-Cyclopenten-1-one, 3-methyl-
9	16.075	144585	38664	3.74	0.07	2,4-Hexadiene, 2,3-dimethyl-
10	16.145	293447	85295	3.44	0.14	2-Cyclopenten-1-one, 2,3-dimethyl-
11	16.239	5573523	1621471	3.44	2.63	Phenol
12	17.566	1361295	747440	1.82	0.64	3-Methylcyclopentane-1,2-dione
13	17.608	4281107	1026401	4.17	2.02	2-Cyclopenten-1-one, 2,3-dimethyl-
14	18.164	92410	44027	2.1	0.04	4-Methyl-5*H*-furan-2-one
15	18.618	92258	49940	1.85	0.04	Bicyclo[2.2.2]octane, 2-methyl-
16	18.68	3854517	958759	4.02	1.82	2-Cyclopenten-1-one, 3-ethyl-2-hydroxy-
17	18.85	4026067	1697344	2.37	1.9	Phenol, 2-methyl-
18	18.935	105210	78437	1.34	0.05	3-Cyclopentene-1,2-diol, cis-
19	18.982	523547	102545	5.11	0.25	Cycloheptanone, 2-methyl-
20	19.091	638719	179199	3.56	0.3	2-Cyclopenten-1-one, 3-ethyl-
21	19.172	204828	126955	1.61	0.1	Cyclohexanol, 2-methyl-, cis-
22	19.205	207050	90857	2.28	0.1	*N*-(2-Butyl)cyclopropanecarboxamide
23	19.322	20663645	7035616	2.94	9.77	Phenol, 2-methoxy-
24	19.535	304003	82339	3.69	0.14	Cyclopentanecarboxaldehyde, 2-methyl-3-methylene-
25	19.671	3778460	1883416	2.01	1.79	p-Cresol
26	19.695	4616530	1788150	2.58	2.18	Phenol, 3-methyl-
27	20.008	83335	50970	1.63	0.04	5-Ethyl-2-furaldehyde
28	20.049	203022	68385	2.97	0.1	2(1*H*)-Pentalenone, 3,3a,4,6a-tetrahydro-
29	20.171	490855	166803	2.94	0.23	Phenol, 2,6-dimethyl-
30	20.312	65551	35581	1.84	0.03	2,3-Dimethyl-4-hydroxy-2-butenoic lactone
31	20.392	175326	63932	2.74	0.08	Levoglucosenone
32	20.841	922192	223207	4.13	0.44	2-Cyclopenten-1-one, 3-ethyl-2-hydroxy-
33	20.958	491552	201703	2.44	0.23	Spiro[2.3]hexan-5-one, 4,4-diethyl-
34	21.619	505352	244283	2.07	0.24	Cyclopentane, 1-methyl-3-(2-methylpropyl)-
35	21.655	507232	211039	2.4	0.24	Phenol, 2-ethyl-
36	21.729	1012607	303130	3.34	0.48	Bicyclo[2.2.2]octan-1-ol, 4-methyl-
37	21.884	2276857	1011817	2.25	1.08	Phenol, 2,4-dimethyl-
38	21.923	1800661	711594	2.53	0.85	Phenol, 2,6-dimethyl-
39	22.048	100399	40857	2.46	0.05	2-Hydroxy-3-propyl-2-cyclopenten-1-one
40	22.218	237639	66242	3.59	0.11	Cantharidin
41	22.33	1503215	640615	2.35	0.71	2-Methoxy-5-methylphenol
42	22.415	48705	32552	1.5	0.02	1H-4-Azacycloprop[cd]indene, octahydro-4-methyl-
43	22.611	3309234	1162174	2.85	1.56	Phenol, 2,3-dimethyl-

44	22.668	670694	263203	2.55	0.32	Phenol, 2-ethyl-
45	22.792	25535138	9746654	2.62	12.07	Creosol
46	23.247	379731	126236	3.01	0.18	Phenol, 2,4,5-trimethyl-
47	23.346	1177276	422469	2.79	0.56	Phenol, 3,4-dimethyl-
48	23.564	288869	108359	2.67	0.14	2-Hydroxy-3-propyl-2-cyclopenten-1-one
49	23.662	2360227	744357	3.17	1.12	1,4:3,6-Dianhydro-.alpha.-d-glucopyranose
50	23.736	1283276	395361	3.25	0.61	cis-3-Methylcyclohexanol
51	23.963	552872	195384	2.83	0.26	2,3-Anhydro-d-mannosan
52	24.05	626885	178189	3.52	0.3	3-Ethylphenol, methyl ether
53	24.217	1488659	501346	2.97	0.7	3-Cyclopentylpropionic acid, 2-isopropoxyphenyl ester
54	24.278	786212	243014	3.24	0.37	Phenol, 2-ethyl-4-methyl-
55	24.365	241445	110251	2.19	0.11	p-Cumenol
56	24.401	461598	151646	3.04	0.22	2,3-Anhydro-d-mannosan
57	24.498	244076	162142	1.51	0.12	5-Hydroxymethylfurfural
58	24.551	1260287	386749	3.26	0.6	Phenol, 2-ethyl-5-methyl-
59	24.598	343639	179761	1.91	0.16	Ethyl 2-cyanocrotonate
60	24.652	508982	178252	2.86	0.24	Phenol, 2-(1-methylethyl)-, methylcarbamate
61	24.708	45272	41249	1.1	0.02	1-Methylverbenol
62	24.745	115783	39860	2.9	0.05	Octanoic acid, silver(1+) salt
63	24.825	694453	178707	3.89	0.33	Phenol, 4-ethyl-2-methoxy-
64	25.082	89457	40552	2.21	0.04	6,9,12-Octadecatrienoic acid, phenylmethyl ester, (Z,Z,Z)-
65	25.227	792084	287236	2.76	0.37	Phenol, 2-ethyl-5-methyl-
66	25.316	11958518	3922072	3.05	5.65	Phenol, 4-ethyl-2-methoxy-
67	25.464	795082	249378	3.19	0.38	Phenol, 2,3,6-trimethyl-
68	25.74	1743303	459775	3.79	0.82	1,2-Benzenediol, 4-methyl-
69	25.902	151932	56550	2.69	0.07	4-Hydroxy-2,4,5-trimethyl-2,5-cyclohexadien-1-one
70	26.02	109433	48711	2.25	0.05	2-Allylphenol
71	26.272	352079	194057	1.81	0.17	3′,5′-Dihydroxyacetophenone
72	26.33	3687077	1366670	2.7	1.74	2-Methoxy-4-vinylphenol
73	26.388	573148	231336	2.48	0.27	3-Dimethylaminomethyleneamino-2-pyridone
74	26.613	1343584	323255	4.16	0.64	1,2-Benzenediol, 4-methyl-
75	26.754	1386948	278816	4.97	0.66	2,6-Dimethoxybenzylamine
76	26.926	204650	66967	3.06	0.1	2-Cyclohexen-1-one, 2-hydroxy-3-methyl-6-(1-methylethyl)-
77	26.972	94616	47633	1.99	0.04	2-Methyl-4-propylphenol
78	27.02	587131	243120	2.41	0.28	3-Methoxy-5-methylphenol
79	27.092	88617	38677	2.29	0.04	Phenol, 2-(1-methylpropyl)-
80	27.178	66062	22009	3	0.03	7-Methylindan-1-one
81	27.259	461868	92482	4.99	0.22	Acetic acid, 2-benzimidazol-2-yl-
82	27.409	654471	242211	2.7	0.31	4-Ethylbenzoic acid, 2-methylpropyl ester
83	27.476	2547367	951054	2.68	1.2	Phenol, 2-methoxy-3-(2-propenyl)-
84	27.554	1244639	417547	2.98	0.59	2-[3-(3,4-Dimethylphenylamino)propionyl]-4-methylphenol
85	27.712	2700464	1003452	2.69	1.28	Phenol, 2-methoxy-4-propyl-
86	27.868	516899	124863	4.14	0.24	Phenol, 3,5-diethyl-
87	28.022	621080	271513	2.29	0.29	3,4-Anhydro-d-galactosan
88	28.068	1485721	437153	3.4	0.7	1,3-Benzenediol, 2,5-dimethyl-
89	28.192	80066	40868	1.96	0.04	Cyclohexane, 1,3-butadienylidene-
90	28.428	156023	65055	2.4	0.07	1H-Indole, 6-methyl-
91	28.512	304962	100373	3.04	0.14	Phenol, 2-methoxy-5-(1-propenyl)-, (E)-
92	28.672	2138211	717790	2.98	1.01	Vanillin

93	28.809	1724969	668630	2.58	0.82	Phenol, 2-methoxy-4-(1-propenyl)-, (*Z*)-
94	28.878	76827	57291	1.34	0.04	Trichloroacetic acid, 2,6-dimethylnon-1-en-3-yn-5-yl ester
95	28.989	176136	50859	3.46	0.08	1,3-Benzenediol, 4-ethyl-
96	29.122	91584	36521	2.51	0.04	3-Octen-5-yne, 2,2,7,7-tetramethyl-
97	29.192	138000	64778	2.13	0.07	(4aS,4bS,10aS)-7-Isopropyl-1,1,4a-trimethyl-1,2,3,4,4a,4b,5,9,10,10a-decahydrophenanthrene
98	29.278	380839	130689	2.91	0.18	3,4-Dimethoxytoluene
99	29.325	139819	71331	1.96	0.07	4-(2,7,7-Trimethylbicyclo[3.2.0]hept-2-en-1-yl)but-3-en-2-one
100	29.488	49696	34178	1.45	0.02	2-Allyl-4-methylphenol
101	29.537	217100	78838	2.75	0.1	3(2H)-Benzofuranone, 7-hydroxy-2,2-dimethyl-
102	29.697	260963	114458	2.28	0.12	1,2-Dimethoxy-4-n-propylbenzene
103	29.838	7141035	2833366	2.52	3.38	Phenol, 2-methoxy-4-(1-propenyl)-, (*Z*)-
104	30.195	152129	74785	2.03	0.07	5,6,7,8-Tetrahydro-2-methyl-1,4-naphthoquinone
105	30.259	296130	103734	2.85	0.14	(*E*)-4-(3-Hydroxyprop-1-en-1-yl)-2-methoxyphenol
106	30.388	765133	126377	6.05	0.36	1,5-Dihydroxy-1,2,3,4-tetrahydronaphthalene
107	30.504	341451	117551	2.9	0.16	3-Methoxycinnamic acid
108	30.664	166648	75904	2.2	0.08	Benzaldehyde, 3,4-dimethoxy-
109	30.715	150681	65408	2.3	0.07	6-Methoxy-3-methylbenzofuran
110	30.843	1150254	443067	2.6	0.54	Apocynin
111	30.925	68887	43635	1.58	0.03	3(2*H*)-Benzofuranone, 7-hydroxy-2,2-dimethyl-
112	30.986	149000	65049	2.29	0.07	Methyleugenol
113	31.108	135781	58032	2.34	0.06	3-Pyridinecarbonitrile, 2-methoxy-4,6-dimethyl-
114	31.198	67908	20533	3.31	0.03	Papaveroline, 1,2,3,4-tetrahydro-
115	31.312	671643	207395	3.24	0.32	2-Amino-4-trifluoromethyl-[1,3]thiazine-6-thione
116	31.362	697146	255440	2.73	0.33	3(2*H*)-Benzofuranone, 6,7-dihydroxy-
117	31.432	1049845	268925	3.9	0.5	Nonanoic acid
118	31.481	458437	273272	1.68	0.22	5-Phenylvaleric acid, 2-ethylhexyl ester
119	31.492	214237	281879	0.76	0.1	Pentaerythritol
120	31.535	619287	282631	2.19	0.29	3-Buten-2-amine, 4-(2,6,6-trimethyl-1-cyclohexen-1-yl)-
121	31.592	1034171	341311	3.03	0.49	2-Amino-2-cyano-4-methylpentanethioamide
122	31.602	1094069	354025	3.09	0.52	2-(3,4-Dimethyl-.alpha.-thiosemicarbazonobenzyl)benzoic acid
123	31.671	979144	363897	2.69	0.46	.beta.-D-Glucopyranose, 1,6-anhydro-
124	31.708	784979	327819	2.39	0.37	.beta.-D-Glucopyranose, 1,6-anhydro-
125	31.772	1859106	397216	4.68	0.88	3,4-Altrosan
126	31.866	3752083	1070075	3.51	1.77	2-Propanone, 1-(4-hydroxy-3-methoxyphenyl)-
127	31.918	2904650	498324	5.83	1.37	.beta.-D-Glucopyranose, 1,6-anhydro-
128	32.069	329587	152984	2.15	0.16	Phenol, 4-ethyl-2-methoxy-
129	32.098	213730	124700	1.71	0.1	*N*-(4-Methoxy-2-nitro-phenyl)-2-(5-methyl-1*H*-benzoimidazol-2-ylsulfanyl)-acetamide
130	32.156	534153	156649	3.41	0.25	2,3-benzofurandimethanol
131	32.659	127234	73632	1.73	0.06	2′,4′-Dimethoxyacetophenone
132	32.824	180558	57560	3.14	0.09	Naphthalene, 1,2-dihydro-7-methoxy-
133	32.898	28962	29631	0.98	0.01	Bicyclo[3.2.0]hept-2-ene, 4-dimethylamino-
134	33.054	561009	181977	3.08	0.27	4-Ethoxy-3-anisaldehyde
135	33.228	271583	115431	2.35	0.13	Diethyl Phthalate
136	33.865	66013	27737	2.38	0.03	*n*-Capric acid isopropyl ester
137	34.25	208381	51134	4.08	0.1	3-*n*-Butylthiane,S-oxide
138	34.455	112173	51797	2.17	0.05	2-Butanone, 4-(4-hydroxy-3-methoxyphenyl)-
139	34.538	59613	32664	1.83	0.03	[1-(4-Fluorophenyl)pyrazol-4-yl]methanol

140	34.565	137490	61981	2.22	0.06	4-Methyl-6-methoxycoumarin
141	34.646	1610204	361005	4.46	0.76	Benzenepropanol, 4-hydroxy-3-methoxy-
142	34.798	456241	129758	3.52	0.22	2-Naphthalenol, 3-methoxy-
143	34.865	28870	18080	1.6	0.01	Estra-1,3,5(10)-trien-17-ol, 3-methoxy-, acetate, (17.beta.)-(.+-.)-
144	35.165	119517	48894	2.44	0.06	5-Amino-6,8-dimethoxyquinoline
145	35.359	145881	46161	3.16	0.07	Benzene, 1,2,3,4-tetramethyl-4-(1-methylethenyl)-
146	35.618	112822	57566	1.96	0.05	Benzofuro[3.2-d]pyrimidin-4(3*H*)-one
147	36.198	120572	50107	2.41	0.06	2-Ethyl-3-methylene-indan-1-one
148	36.282	153583	55544	2.77	0.07	*N*-[(7-Methoxy-2*H*-1,3-benzodioxol-5-yl)methylidene]hydroxylamine
149	36.382	82144	36558	2.25	0.04	Coniferyl aldehyde
150	36.581	49136	31816	1.54	0.02	silane, trimethyl[[5-methyl-2-(1-methylethyl)-1,3-dioxan-5-yl]methoxy]-
151	36.685	38290	12847	2.98	0.02	Astaxanthin
152	36.851	80895	36405	2.22	0.04	2,5-Dimethyl-3-phenylfuran
153	37.105	472034	134876	3.5	0.22	2-Acetyl-1-tetralone
154	37.245	86893	35226	2.47	0.04	1-Octadecanesulphonyl chloride
155	37.306	172125	58276	2.95	0.08	Benzenesulfonamide, *N*-butyl-
156	37.687	165343	66720	2.48	0.08	Propanehydrazide, N2-(4-methoxyphenyl)-
157	38.087	180508	46160	3.91	0.09	.alpha.-Dehydro-ar-himachalene
158	40.182	91912	49494	1.86	0.04	Pyrrolo[1,2-a]pyrazine-1,4-dione, hexahydro-3-(2-methylpropyl)-
159	40.413	105558	43698	2.42	0.05	5,10-Diethoxy-2,3,7,8-tetrahydro-1*H*,6H-dipyrrolo[1,2-a:1′,2′-d]pyrazine
160	40.615	102717	55545	1.85	0.05	Benzene, 1-(2,2-dichloro-1-methylcyclopropyl)-4-nitro-
161	42.139	83842	35402	2.37	0.04	6-(1,3-Dimethyl-buta-1,3-dienyl)-1,5,5-trimethyl-7-oxa-bicyclo[4.1.0]hept-2-ene
162	42.258	326728	110194	2.97	0.15	Phenanthrene, 2,5-dimethyl-
163	43.191	107147	39559	2.71	0.05	10,18-Bisnorabieta-5,7,9(10),11,13-pentaene
164	44.471	228634	74616	3.06	0.11	Phenanthrene, 2,3,5-trimethyl-
165	44.895	155648	65161	2.39	0.07	Dichloroacetic acid, 3-pentadecyl ester
166	45.088	69901	37906	1.84	0.03	Nonadecanamide
167	45.634	863137	326513	2.64	0.41	Retene

Table S3. GC-MS data for Cat_C.

Peak#	Ret.Time	Area	Height	A/H	Conc.	Name
1	1.785	645204	419088	1.54	0.08	Formic acid
2	1.855	1647664	1585859	1.04	0.21	Methanamine, *N*-hydroxy-N-methyl-
3	1.962	412989	326263	1.27	0.05	3-Pentanone
4	2.071	783912	502033	1.56	0.1	Lactic acid
5	2.214	4601758	2376232	1.94	0.57	Acetic acid
6	2.433	8049688	3749678	2.15	1	2-Propanone, 1-hydroxy-
7	3.448	307277	108333	2.84	0.04	Thiazolidin-4-one, 5-ethyl-2-imino-
8	3.511	509592	181308	2.81	0.06	Propanoic acid
9	4.086	784732	257844	3.04	0.1	1-Hydroxy-2-butanone
10	4.173	2788607	654741	4.26	0.35	Acetic acid, methyl ester
11	4.288	1531915	352762	4.34	0.19	Propanal
12	5.989	770844	183634	4.2	0.1	Butanoic acid, 2-propenyl ester
13	6.223	744458	321402	2.32	0.09	Furfural

14	6.264	2257517	353301	6.39	0.28	2-Cyclopenten-1-one
15	6.838	450715	257976	1.75	0.06	2-Pentanone, 4-hydroxy-4-methyl-
16	6.874	1205987	268979	4.48	0.15	Butanoic acid, 3-hydroxy-3-methyl-
17	6.965	301368	127009	2.37	0.04	1,3-Dioxolane-4-carboxaldehyde, 2,2-dimethyl-, (*R*)-
18	7.969	1617471	286291	5.65	0.2	2-Furanmethanol
19	12.513	1040669	241840	4.3	0.13	1,2-Cyclopentanedione
20	12.595	295093	151989	1.94	0.04	Piperidine, 2,3-dimethyl-
21	13.986	1140830	435510	2.62	0.14	2-Furancarboxaldehyde, 5-methyl-
22	14.032	461319	395119	1.17	0.06	2-Butyn-1-yl chloroformate
23	14.045	494568	363475	1.36	0.06	2-Ethoxypyridine *N*-oxide
24	14.068	1476458	355839	4.15	0.18	2-Furancarboxaldehyde, 5-methyl-
25	14.245	236124	181497	1.3	0.03	Propanoic acid, ethenyl ester
26	14.28	1452926	303249	4.79	0.18	2-Cyclopenten-1-one, 3-methyl-
27	14.395	320319	146223	2.19	0.04	Isobutyl prop-2-ynyl carbonate
28	14.415	239678	118021	2.03	0.03	Acetamide, 2,2,2-trifluoro-*N*-(1-methylpiperidin-4-yl)-
29	14.492	670739	177930	3.77	0.08	2-Cyclopenten-1-one, 3-methyl-
30	16.225	8913524	2588310	3.44	1.11	Phenol
31	16.422	576161	150949	3.82	0.07	3-Trifluoroacetoxypentadecane
32	16.514	507888	125837	4.04	0.06	4-Methyl -2*H*-pyrazole-3-carbaldehyde
33	17.259	440945	132456	3.33	0.05	4-Methyl -1*H*-pyrazole-3,5-diamine
34	17.525	15975271	3811296	4.19	1.99	2-Cyclopenten-1-one, 2-hydroxy-3-methyl-
35	17.86	832858	215917	3.86	0.1	4-Methyl-5*H*-furan-2-one
36	18.488	211064	97756	2.16	0.03	Bicyclo[2.2.2]octane, 2-methyl-
37	18.643	4074260	1208778	3.37	0.51	2-Cyclopenten-1-one, 3-ethyl-2-hydroxy-
38	18.854	9688923	2814467	3.44	1.21	Phenol, 2-methyl-
39	19.113	249790	134527	1.86	0.03	1-Penten-3-one, 2,4-dimethyl-
40	19.336	45248347	12845814	3.52	5.64	Phenol, 2-methoxy-
41	19.688	14870729	2927681	5.08	1.85	Phenol, 3-methyl-
42	19.936	350885	99199	3.54	0.04	Cyclohexane, (1-methylethylidene)-
43	20.146	2744871	575041	4.77	0.34	Phenol, 2,6-dimethyl-
44	20.27	1025162	239621	4.28	0.13	Levoglucosenone
45	20.61	213748	53073	4.03	0.03	1,2,3-Benzenetriol
46	20.748	2935861	887218	3.31	0.37	2-Cyclopenten-1-one, 3-ethyl-2-hydroxy-
47	20.871	1348917	370883	3.64	0.17	Spiro[2.3]hexan-5-one, 4,4-diethyl-
48	21.037	462790	119131	3.88	0.06	2-Methoxy-6-methylphenol
49	21.114	480556	87204	5.51	0.06	1,3-Dioxolane, 2-(3-bromo-3-buten-1-yl)-
50	21.242	216517	70417	3.07	0.03	4-Hexen-3-one, 4-methyl-
51	21.403	804550	224329	3.59	0.1	4,6-Dimethyl-2-pyrimidinylhydrazine
52	21.488	261297	98134	2.66	0.03	1,3-Hexadiene, 3-ethyl-2,5-dimethyl-
53	21.569	982483	332722	2.95	0.12	1-Oxetan-2-one, 4,4-diethyl-3-methylene-
54	21.69	1937286	376868	5.14	0.24	Bicyclo[2.2.1]heptan-2-ol
55	21.882	6818139	1593474	4.28	0.85	Phenol, 2,4-dimethyl-
56	22.182	211375	69981	3.02	0.03	2,3a-Dimethylhexahydrobenzofuran-7a-ol
57	22.224	229548	79824	2.88	0.03	Hexanoic acid, 2-propenyl ester
58	22.312	2892974	1118205	2.59	0.36	Creosol
59	22.604	4625975	1441855	3.21	0.58	Phenol, 3,4-dimethyl-
60	22.668	939121	439222	2.14	0.12	Phenol, 2-ethyl-
61	22.816	57264615	17819086	3.21	7.13	Creosol
62	22.976	540843	141890	3.81	0.07	trans-3,4-Epoxynonane

63	23.088	671389	121864	5.51	0.08	Heptanal
64	23.233	883730	257823	3.43	0.11	Phenol, 2,4,6-trimethyl-
65	23.341	2101368	663314	3.17	0.26	Phenol, 3,4-dimethyl-
66	23.448	543207	145945	3.72	0.07	1,3-Dioxolane-4-ethanol, 2,2,4-trimethyl-
67	23.522	765044	219401	3.49	0.1	2-Hydroxy-3-propyl-2-cyclopenten-1-one
68	23.628	463935	111258	4.17	0.06	3H-Pyrazol-3-one, 2,4-dihydro-4,4,5-trimethyl-
69	23.771	9821184	1586239	6.19	1.22	1,4:3,6-Dianhydro-.alpha.-d-glucopyranose
70	23.989	4368084	899202	4.86	0.54	2,3-Anhydro-d-mannosan
71	24.197	17146455	2920157	5.87	2.14	Catechol
72	24.559	21876462	2534029	8.63	2.73	5-Hydroxymethylfurfural
73	24.805	1500898	443677	3.38	0.19	Benzene, 1,4-dimethoxy-2-methyl-
74	24.926	3294598	324455	10.15	0.41	1,2,3,4-Cyclopentanetetrol, (1.alpha.,2.beta.,3.beta.,4.alpha.)-
75	25.128	857695	214073	4.01	0.11	1,2-Benzenediol, 3-methoxy-
76	25.316	21345789	6477155	3.3	2.66	Phenol, 4-ethyl-2-methoxy-
77	25.456	1275684	392364	3.25	0.16	Phenol, 2,4,6-trimethyl-
78	25.734	6591897	1582608	4.17	0.82	1,2-Benzenediol, 3-methyl-
79	25.876	1196491	257115	4.65	0.15	1-Cyclohexene-1-carboxaldehyde, 2,6,6-trimethyl-
80	25.973	2206575	247940	8.9	0.27	1H-Inden-1-one, 2,3-dihydro-3-methyl-
81	26.168	831546	169305	4.91	0.1	4-(3,7,7-Trimethyl-2-oxabicyclo[3.2.0]hept-3-en-1-yl)but-3-en-2-one
82	26.324	7673609	2285575	3.36	0.96	2-Methoxy-4-vinylphenol
83	26.503	2773144	375233	7.39	0.35	Hydroquinone
84	26.63	7033687	1367944	5.14	0.88	1,2-Benzenediol, 4-methyl-
85	26.735	3924637	678331	5.79	0.49	ethanone, 1,1′-(1,2,3,4-tetramethyl-3-cyclobutene-1,2-diyl)bis-
86	26.875	1349908	285800	4.72	0.17	Cyclohexanecarbonitrile, 3,3,5,5-tetramethyl-1-(phenylsulfinyl)-
87	27.016	1544624	338767	4.56	0.19	3-Methoxy-5-methylphenol
88	27.075	728027	199223	3.65	0.09	Acetic acid, [(phenylthioxomethyl)thio]-
89	27.175	363883	119633	3.04	0.05	Cinnamaldehyde, .beta.-methyl-
90	27.296	1174173	229444	5.12	0.15	2,3-*O*-Benzal-d-mannosan
91	27.403	1420701	478163	2.97	0.18	Phenol, 3-methoxy-2,4,5-trimethyl-
92	27.468	5197564	2037292	2.55	0.65	Phenol, 2-methoxy-3-(2-propenyl)-
93	27.544	2351853	729266	3.22	0.29	Benzaldehyde, 2-hydroxy-3-(2-propenyl)-
94	27.628	436975	155602	2.81	0.05	1,4-Benzenedicarboxaldehyde, 2-methyl-
95	27.703	4863912	1883436	2.58	0.61	Phenol, 2-methoxy-4-propyl-
96	27.851	1257651	196330	6.41	0.16	Phenol, 2,3,5,6-tetramethyl-
97	28.08	4808946	808548	5.95	0.6	Creosol
98	28.259	1209949	205541	5.89	0.15	7-Methylindan-1-one
99	28.402	413883	107998	3.83	0.05	3-Methyl-4-(3,7,7-trimethyl-2-oxa-bicyclo[3.2.0]hept-3-en-1-yl)-but-3-en-2-one
100	28.499	954720	188270	5.07	0.12	Phenol, 2-methoxy-4-(1-propenyl)-
101	28.684	10997147	3381444	3.25	1.37	Vanillin
102	28.799	3844887	1373762	2.8	0.48	Phenol, 2-methoxy-4-(1-propenyl)-, (Z)-
103	28.933	969190	195808	4.95	0.12	2-Propanone, 1-(4-hydroxy-3-methoxyphenyl)-
104	29.009	1767446	381659	4.63	0.22	4-Ethylcatechol
105	29.115	520165	178972	2.91	0.06	Phenol, 2-methoxy-6-(2-propenyl)-
106	29.183	997529	273905	3.64	0.12	Propanamide, N-(2,6-dimethylphenyl)-2-[(3,4,5,6-tetrahydro-2H-azepin-7-yl)amino]-

107	29.262	1719049	322967	5.32	0.21	4-Hydroxy-2,6,6-trimethyl-3-oxocyclohexa-1,4-dienecarbaldehyde
108	29.375	385008	113604	3.39	0.05	2-Amino-4-(tert-butyl)thiophene-3-carbonitrile
109	29.553	1736201	217560	7.98	0.22	1,4-Benzenediol, 2,6-dimethyl-
110	29.689	865786	286380	3.02	0.11	1,2-Dimethoxy-4-n-propylbenzene
111	29.836	15799772	6387714	2.47	1.97	Phenol, 2-methoxy-4-(1-propenyl)-, (*Z*)-
112	29.93	516757	162501	3.18	0.06	Bicyclo[2.2.1]heptane, 2-cyclopropylidene-1,7,7-trimethyl-
113	30.041	880914	151129	5.83	0.11	Creosol
114	30.135	1048132	345222	3.04	0.13	Phenol, 2-methoxy-4-propyl-
115	30.188	562887	247615	2.27	0.07	Benzofuran, 5-methoxy-6,7-dimethyl-
116	30.263	738365	191463	3.86	0.09	Phenol, 2-methoxy-6-(2-propenyl)-
117	30.348	1484903	200104	7.42	0.18	4-Methoxy-7-methylindan-1-one
118	30.485	1176395	264483	4.45	0.15	2-Allyl-1,4-dimethoxybenzene
119	30.649	364535	124841	2.92	0.05	Benzaldehyde, 3,4-dimethoxy-
120	30.704	273512	106832	2.56	0.03	1-(4-Hydroxybenzylidene)acetone
121	30.845	6332671	2318461	2.73	0.79	Apocynin
122	30.967	356802	136621	2.61	0.04	Methyleugenol
123	31.087	931278	157712	5.9	0.12	Benzaldehyde, 3,4-dimethoxy-
124	31.195	928058	171230	5.42	0.12	2-(1,4,4-Trimethylcyclohex-2-en-1-yl)ethyl p-toluenesulfonate
125	31.29	932630	228236	4.09	0.12	2,4-Dimethoxybenzyl alcohol
126	31.434	403094	89657	4.5	0.05	1,3,7,7-Tetramethyl-2,11-dioxa-bicyclo[4.4.1]undeca-3,5,9-trien-8-one
127	31.592	1783886	443868	4.02	0.22	Benzoic acid, 4-hydroxy-3-methoxy-, methyl ester
128	31.78	2287097	388228	5.89	0.28	.beta.-Methylcinnamic acid
129	31.856	8621886	2419754	3.56	1.07	2-Propanone, 1-(4-hydroxy-3-methoxyphenyl)-
130	31.985	2460271	518339	4.75	0.31	Carbaril
131	32.147	6507057	728407	8.93	0.81	2,3-benzofurandimethanol
132	32.343	12002501	1205024	9.96	1.5	1-Nitro-.beta.-d-arabinofuranose, tetraacetate
133	32.496	2541332	832395	3.05	0.32	.alpha.-D-Glucopyranose, 4-*O*-.beta.-D-galactopyranosyl-
134	32.522	2164288	784632	2.76	0.27	2,6-Octadienoic acid, 4-isopropylidene-6,7-dimethyl-, methyl ester
135	32.588	2528033	836221	3.02	0.31	.beta.-D-Glucopyranose, 1,6-anhydro-
136	32.643	3489491	947023	3.68	0.43	5-Chloro-2-(3,4-dimethoxybenzamido)benzoic acid
137	32.718	3296352	983070	3.35	0.41	.beta.-D-Glucopyranose, 1,6-anhydro-
138	32.824	9921970	1490769	6.66	1.24	4-(1-Hydroxyallyl)-2-methoxyphenol
139	32.901	1613081	1021789	1.58	0.2	Dimethylmalonic acid, 2-ethylphenyl heptyl ester
140	32.922	1610745	1015762	1.59	0.2	D-Allose
141	33.043	12393023	1752140	7.07	1.54	1-(2-Hydroxy-4-methoxyphenyl)propan-1-one
142	33.171	13723210	1796514	7.64	1.71	Butyrovanillone
143	33.355	8473707	1370682	6.18	1.06	Pentane, 1,1,1,5-tetrachloro-
144	33.375	2223596	1421744	1.56	0.28	.beta.-D-Glucopyranose, 1,6-anhydro-
145	33.433	5559913	1455849	3.82	0.69	3,4-Altrosan
146	33.504	7031515	1543751	4.55	0.88	D-Allose
147	33.588	7953149	1581638	5.03	0.99	Pentanoic acid
148	33.781	22702857	1955223	11.61	2.83	3,4-Altrosan
149	33.902	8115750	1875693	4.33	1.01	Nonanoic acid
150	33.954	7739025	2012649	3.85	0.96	Benzamide, 4-butoxy-*N*-[2-(2-thienyl)ethyl]-
151	34.029	13822098	2097517	6.59	1.72	Propane, 1-(ethenylthio)-
152	34.11	5836253	2147312	2.72	0.73	.beta.-D-Ribopyranoside, methyl

153	34.258	37314523	2258866	16.52	4.65	.beta.-D-Glucopyranose, 1,6-anhydro-
154	34.665	6354808	1402589	4.53	0.79	Benzenepropanol, 4-hydroxy-3-methoxy-
155	34.798	1979441	513877	3.85	0.25	2-Naphthalenol, 3-methoxy-
156	34.944	527992	132315	3.99	0.07	1-(2-Hydroxyadamantan-1-yl)ethanone
157	35.063	528281	101383	5.21	0.07	2-(2,5-Dimethoxy-phenyl)-propionaldehyde
158	35.202	711416	117348	6.06	0.09	4-(1-Hydroxyallyl)-2-methoxyphenol
159	35.49	701807	196163	3.58	0.09	Phenylacetylformic acid, 4-hydroxy-3-methoxy-
160	35.715	4305890	301645	14.27	0.54	1,6-Anhydro-.beta.-D-glucofuranose
161	36.263	297900	102797	2.9	0.04	2-Cyclohexen-1-one, 3,5,5-trimethyl-, semicarbazone
162	36.376	1907771	705783	2.7	0.24	Coniferyl aldehyde
163	36.682	351823	96353	3.65	0.04	3,4-2*H*-Coumarin, 4,4,5,6,8-pentamethyl-
164	36.833	378537	78668	4.81	0.05	4-(Methylthio)benzoic acid, 2-(2-chlorophenoxy)ethyl ester
165	37.089	1185127	356210	3.33	0.15	Naphthalene, 2,3-dimethoxy-
166	37.18	393698	122698	3.21	0.05	Benzenemethanol, .alpha.-1-pentynyl-
167	37.303	321377	78766	4.08	0.04	Benzenesulfonamide, N-butyl-
168	37.452	316936	74923	4.23	0.04	Glutaric acid, 3-methylbut-2-en-1-yl 4-(4-methoxyphenyl)cyclohexyl ester
169	37.684	354357	141543	2.5	0.04	4-Hydroxy-3-methoxybenzyl alcohol, di(pentyl) ether
170	37.875	319816	120601	2.65	0.04	3-Ethoxycarbonyl-5,5-dimethyl-1-pyrroline, 1-oxide
171	38.022	308660	90186	3.42	0.04	3-Buten-2-one, 4-(4-hydroxy-3-methoxyphenyl)-
172	38.083	330409	128811	2.57	0.04	2-Naphthaleneacetaldehyde, 1,4-dihydro-.alpha.,.alpha.-dimethyl-1,4-dioxo-
173	38.136	374237	133483	2.8	0.05	1-Nonanol
174	38.203	397628	108895	3.65	0.05	Coniferyl aldehyde
175	38.284	714608	149442	4.78	0.09	1,3-Dioxan-4-one, 6-methyl-2-octyl-, (2R-cis)-
176	38.469	305371	77751	3.93	0.04	2-Allyl-3,6-dimethoxybenzyl alcohol
177	38.575	328214	60025	5.47	0.04	Cyclohepta[b]naphthalene-1-one
178	38.801	428279	122899	3.48	0.05	Dimethylmalonic acid, monochloride, nonyl ester
179	39.338	296568	46563	6.37	0.04	(.+/-.)-Tremetone
180	39.516	315852	70160	4.5	0.04	[2-(4-Methoxyphenyl)-5-oxocyclopent-1-enyl]acetic acid
181	40.013	355793	105050	3.39	0.04	(.+/-.)-Tremetone
182	40.621	574183	184404	3.11	0.07	4,4,5,8-Tetramethyl-chroman-2-one
183	40.755	205133	45806	4.48	0.03	Lumazine, 8-ethyl-6,7-dimethyl-
184	40.904	238292	56580	4.21	0.03	D-Norandrostan-16-one, (5.alpha.)-
185	41.104	504859	64682	7.81	0.06	2,7-Dimethyldibenzothiophene
186	41.362	250833	43269	5.8	0.03	Azuleno[4,5-b]furan-2(3H)-one, decahydro-7,9-dihydroxy-6,9a-dimethyl-3-methylene-, [3aS-(3a.alpha.,6.beta.,6a.alpha.,7.alpha.,9
187	41.669	290441	75931	3.83	0.04	4b,8-Dimethyl-2-isopropylphenanthrene, 4b,5,6,7,8,8a,9,10-octahydro-
188	41.735	316292	83955	3.77	0.04	12,13-Dioxatricyclo[7.3.1.0(1,6)]tridecane-8-carboxylic acid, 6-methyl-5-([(4-methylphenyl)sulfonyl]oxy)
189	41.868	290552	52713	5.51	0.04	4H-Pyran-4-one, 2-methoxy-3,5-dimethyl-6-(tetrahydro-4-hydroxy-2-furyl)-
190	41.986	348692	78217	4.46	0.04	Benzene, 1,1′-methylenebis[3-methyl-
191	42.136	331966	99290	3.34	0.04	5-(4-Methoxy-benzylidene)-imidazolidine-2,4-dione
192	42.249	531455	130436	4.07	0.07	Phenanthrene, 2,5-dimethyl-
193	42.355	255773	55549	4.6	0.03	benzo[b]thiophene-5-carboxylic acid, methyl ester, 1,1-dioxide

194	42.456	382348	92593	4.13	0.05	Naphthalene, 6-methoxy-2-(1-buten-3-yl)-
195	42.673	506700	67828	7.47	0.06	2-tert-Butyl-5-(3-methoxy-benzylidene)-6-methyl-[1,3]dioxan-4-one
196	42.769	674589	69742	9.67	0.08	trans-Bicyclo[4.3.0]nonane, 1-methyl-9-(2-octyl)-5-(2-trimethylsilyloxy)-4-(1-methyl-2,4 -di(trimethylsilyloxy)cyclohexyl-
197	43.165	364692	73562	4.96	0.05	4,4′-Diisopropylbiphenyl
198	43.537	482801	58689	8.23	0.06	Cycloprop[e]indene-1a,2(1*H*)-dicarboxaldehyde, 3a,4,5,6,6a,6b-hexahydro-5,5,6b-trimethyl-, (1a.alpha.,3a.beta.,6a.beta.,6b.alpha
199	44.46	507503	155288	3.27	0.06	Succinic acid, 4-cyanophenyl 3,4-dimethylphenyl ester
200	44.78	211140	53506	3.95	0.03	Coumarine, 8-allyl-7-hydroxy-6-ethyl-4-methyl-
201	44.889	292914	112416	2.61	0.04	1-Octadecanol
202	45.629	1002567	350050	2.86	0.12	Retene

Table S4. GC-MS data for Cat_D.

Peak#	Ret.Time	Area	Height	A/H	Conc.	Name
1	1.801	181619	194500	0.93	0.07	1,3-Butanediol
2	2.099	186880	109965	1.7	0.07	Isopropyl Alcohol
3	2.184	1575913	1210570	1.3	0.59	Acetic acid
4	3.451	208522	90274	2.31	0.08	Propanoic acid
5	6.142	137740	49040	2.81	0.05	1,6-Heptadien-4-ol
6	6.243	211701	55662	3.8	0.08	Butanoic acid
7	6.36	179783	95641	1.88	0.07	Furfural
8	13.942	125092	34672	3.61	0.05	2*H*-Pyran-2-one, tetrahydro-
9	14.056	677407	227010	2.98	0.26	2-Furancarboxaldehyde, 5-methyl-
10	14.125	23398	44871	0.52	0.01	Ethyl trans-3-methyltetrazole-5-acrylate
11	14.395	510928	150792	3.39	0.19	2-Cyclopenten-1-one, 3-methyl-
12	14.487	232691	99308	2.34	0.09	2,4-Dimethylfuran
13	16.213	4992023	1403836	3.56	1.88	Phenol
14	17.445	2640024	1002603	2.63	0.99	2-Cyclopenten-1-one, 2-hydroxy-3-methyl-
15	17.497	4045627	1144971	3.53	1.52	2-Cyclopenten-1-one, 2,3-dimethyl-
16	17.628	111054	128060	0.87	0.04	2-(3-Fluorophenyl)-6-piperidino-4,4-bis(trifluoromethyl)-1,3,5-oxadiazine
17	17.675	276674	87396	3.17	0.1	Bicyclobutylidene
18	17.705	80257	61551	1.3	0.03	Uracil
19	17.989	131178	90876	1.44	0.05	2-Hexenal, (*E*)-
20	18.028	68390	43412	1.58	0.03	1*H*-[1,2,4]Triazole-3-carboxylic acid (2-cyclohex-1-enyl-ethyl)-amide
21	18.518	207998	45029	4.62	0.08	2-Cyclopenten-1-one, 3,4,4-trimethyl-
22	18.594	3048004	738941	4.12	1.15	2-Cyclopenten-1-one, 3-ethyl-2-hydroxy-
23	18.83	4056980	1756596	2.31	1.53	Phenol, 2-methyl-
24	18.948	567011	157627	3.6	0.21	Bicyclo[3.3.1]nonane
25	19.008	448684	153484	2.92	0.17	2-Cyclopenten-1-one, 3-ethyl-
26	19.095	226056	54487	4.15	0.09	2-Cyclopenten-1-one, 3-ethyl-
27	19.303	20974535	7432860	2.82	7.9	Phenol, 2-methoxy-
28	19.668	7904764	1727857	4.57	2.98	Phenol, 3-methyl-
29	19.825	155728	37723	4.13	0.06	Cyclohexanone, 3-ethenyl-
30	20.154	635529	228242	2.78	0.24	Phenol, 2,6-dimethyl-
31	20.238	26698	19868	1.34	0.01	5-Octen-2-one, 3,6-dimethyl-
32	20.345	68483	46698	1.47	0.03	Levoglucosenone

33	20.635	58214	21945	2.65	0.02	Benzofurazan, 4,5,6,7-tetrahydro-
34	20.754	848463	223967	3.79	0.32	2-Cyclopenten-1-one, 3-ethyl-2-hydroxy-
35	20.835	147895	85960	1.72	0.06	Pyrimidine-4,6-diol, 5-methyl-
36	20.883	473269	157633	3	0.18	Spiro[2.3]hexan-5-one, 4,4-diethyl-
37	21.415	196676	78569	2.5	0.07	Benzene, 1,2-dimethoxy-
38	21.541	520744	183685	2.83	0.2	Bicyclo[2.2.2]octan-1-ol, 2-methyl-
39	21.66	868943	289298	3	0.33	Bicyclo[2.2.2]octan-1-ol, 4-methyl-
40	21.867	2358370	1032275	2.28	0.89	Phenol, 2,4-dimethyl-
41	21.908	1691967	706229	2.4	0.64	Phenol, 2,6-dimethyl-
42	22.148	114094	46145	2.47	0.04	5-Ethyl-2-furaldehyde
43	22.206	229914	75575	3.04	0.09	Azulene
44	22.314	1526761	588433	2.59	0.58	2-Methoxy-5-methylphenol
45	22.591	3256721	1206733	2.7	1.23	Phenol, 3,4-dimethyl-
46	22.658	853219	321301	2.66	0.32	Phenol, 3-ethyl-
47	22.779	28816428	10833736	2.66	10.86	Creosol
48	22.952	149993	37199	4.03	0.06	Furan, tetrahydro-2-isopentyl-5-propyl-
49	23.071	124370	48619	2.56	0.05	Benzoic acid, 2-(2-oxo-2-piperidin-1-yl-ethylsulfanyl)-
50	23.234	265530	126606	2.1	0.1	Phenol, 2,3,5-trimethyl-
51	23.327	1175395	476884	2.46	0.44	Phenol, 3,4-dimethyl-
52	23.508	134754	76672	1.76	0.05	2-Hydroxy-3-propyl-2-cyclopenten-1-one
53	23.642	2414873	740667	3.26	0.91	1,4:3,6-Dianhydro-.alpha.-d-glucopyranose
54	23.712	1729380	518403	3.34	0.65	2,3-Anhydro-d-galactosan
55	23.931	868201	287841	3.02	0.33	2,3-Anhydro-d-mannosan
56	24.038	556657	139739	3.98	0.21	Phenol, 2,3,6-trimethyl-
57	24.159	4631197	1096205	4.22	1.75	Catechol
58	24.265	730241	260499	2.8	0.28	Phenol, 3-ethyl-5-methyl-
59	24.352	293955	109707	2.68	0.11	Phenol, 2,3,6-trimethyl-
60	24.428	3383553	876509	3.86	1.27	5-Hydroxymethylfurfural
61	24.533	2165989	521177	4.16	0.82	Phenol, 2-ethyl-5-methyl-
62	24.638	778327	212694	3.66	0.29	Phenol, 2-ethyl-4-methyl-
63	24.815	633493	203894	3.11	0.24	Benzene, 1,4-dimethoxy-2-methyl-
64	25.028	103943	56780	1.83	0.04	Benzene, 1-methoxy-2-(methoxymethyl)-
65	25.108	115156	41178	2.8	0.04	1,2-Benzenediol, 3-methoxy-
66	25.205	860510	305335	2.82	0.32	Phenol, 3-ethyl-5-methyl-
67	25.303	11590688	3779643	3.07	4.37	Phenol, 4-ethyl-2-methoxy-
68	25.447	488485	221406	2.21	0.18	Phenol, 2,3,5-trimethyl-
69	25.714	3021602	901352	3.35	1.14	1,2-Benzenediol, 3-methyl-
70	25.975	223143	77968	2.86	0.08	Naphthalene, 1,2,3,4-tetrahydro-6-methyl-
71	26.162	66239	36175	1.83	0.02	Phenol, 4-(aminomethyl)-2-methoxy-
72	26.252	487981	183288	2.66	0.18	3′,5′-Dihydroxyacetophenone
73	26.32	3680688	1242164	2.96	1.39	2-Methoxy-4-vinylphenol
74	26.583	2904511	778275	3.73	1.09	1,2-Benzenediol, 4-methyl-
75	26.732	1375423	337310	4.08	0.52	Benzene, 1-methoxy-2-(methoxymethyl)-
76	26.868	142356	41060	3.47	0.05	2-Cyclohexen-1-one, 2-hydroxy-3-methyl-6-(1-methylethyl)-
77	27.003	681020	236259	2.88	0.26	3-Methoxy-5-methylphenol
78	27.085	199569	42027	4.75	0.08	((1*R*,3*R*)-2,2-dimethyl-3-(2-methylprop-1-en-1-yl)cyclopropyl)methyl acetate
79	27.258	582457	111603	5.22	0.22	1-(Alpha-aminotoluene-p-sulfonyl)-3-butylurea
80	27.401	811047	234710	3.46	0.31	Ethanone, 1-(2-hydroxy-5-methoxyphenyl)-

81	27.462	2493350	1016478	2.45	0.94	Phenol, 2-methoxy-3-(2-propenyl)-
82	27.537	1295343	427315	3.03	0.49	1-(2,4-Dimethoxyphenyl)-3-(3,4-dimethoxyphenyl)prop-2-en-1-one
83	27.615	122927	46304	2.65	0.05	Pyrrole-3-carbonitrile, 5-formyl-2,4-dimethyl-
84	27.699	2673671	1047865	2.55	1.01	Phenol, 2-methoxy-4-propyl-
85	27.843	434426	114775	3.79	0.16	2,5-Diethylphenol
86	27.895	27553	40642	0.68	0.01	2-Methylpiperidine-1-thiocarboxylic acid 2-[1-[2-pyridyl 1-oxide]
87	28.008	766515	345971	2.22	0.29	1,4-Benzenediol, 2-methyl-
88	28.049	1661552	485453	3.42	0.63	1,3-Benzenediol, 2,5-dimethyl-
89	28.142	201790	66080	3.05	0.08	1-Ethyl-3-nitrobenzene
90	28.182	11695	27658	0.42	0	Butane, 1-(2,2-dichloro-3-ethylcyclopropyl)-
91	28.25	76637	40058	1.91	0.03	Tricyclo[5.2.1.0(2,6)]deca-4,8-dien-3-one
92	28.648	4129258	1258232	3.28	1.56	Vanillin
93	28.801	2013480	707282	2.85	0.76	Phenol, 2-methoxy-4-(1-propenyl)-, (*Z*)-
94	28.861	623375	225928	2.76	0.23	2,3-Dimethylhydroquinone
95	28.928	199227	117066	1.7	0.08	Isoquinoline-1-carbonitrile, 2-hex-4-ynoyl-1,2-dihydro-
96	28.971	820903	203032	4.04	0.31	4-Ethylcatechol
97	29.115	319637	64110	4.99	0.12	2-Phenylthiolane
98	29.208	441536	117950	3.74	0.17	17-(2-Hydroxy-1-oxoethyl)-3-salicyloylhydrazono-4-estrene
99	29.258	448320	177147	2.53	0.17	2-Allyl-4-methylphenol
100	29.318	184287	77887	2.37	0.07	2-Allyl-4-methylphenol
101	29.388	100390	40740	2.46	0.04	*m*-Phenylenediamine, TMS derivative
102	29.515	288789	104232	2.77	0.11	1,3-Benzenediol, 4,5-dimethyl-
103	29.684	236892	106297	2.23	0.09	1,2-Dimethoxy-4-*n*-propylbenzene
104	29.823	7803147	3105267	2.51	2.94	Phenol, 2-methoxy-4-(1-propenyl)-, (*Z*)-
105	29.945	187832	73890	2.54	0.07	1-(9-Allyl-9-azabicyclo[3.3.1]non-3-yl)-3-*m*-tolylurea
106	29.998	260623	72392	3.6	0.1	1,3-Benzenediol, 4-ethyl-
107	30.055	87131	44069	1.98	0.03	6-Methyl-4-indanol
108	30.191	242702	94890	2.56	0.09	Benzofuran, 5-methoxy-6,7-dimethyl-
109	30.304	567092	127098	4.46	0.21	2-Isopropylbenzenethiol, S-acetyl-
110	30.365	608844	124032	4.91	0.23	1,5-Dihydroxy-1,2,3,4-tetrahydronaphthalene
111	30.477	446971	109031	4.1	0.17	Methyleugenol
112	30.642	114447	61158	1.87	0.04	Benzaldehyde, 3,4-dimethoxy-
113	30.708	115998	45333	2.56	0.04	6-Methoxy-3-methylbenzofuran
114	30.818	1970465	797244	2.47	0.74	Apocynin
115	30.898	84771	38681	2.19	0.03	2-Cyclopenten-1-one, 4-hydroxy-3-methyl-2-(2,4-pentadienyl)-, (*Z*)-(+)-
116	30.97	102632	51826	1.98	0.04	Benzene, 1,2-dimethoxy-4-(1-propenyl)-
117	31.178	93576	39176	2.39	0.04	(2-Ethoxyphenyl)hydrazine
118	31.345	1078046	225559	4.78	0.41	1-Allyl-3-(2-hydroxyethyl)-2-thiourea
119	31.412	908664	255467	3.56	0.34	Cedran-diol, 8S,13-
120	31.449	559017	270096	2.07	0.21	Morpholine, 4-methyl-, 4-oxide
121	31.585	2964204	423545	7	1.12	Benzoic acid, 4-hydroxy-3-methoxy-, methyl ester
122	31.675	1046567	332907	3.14	0.39	*N*-(4-Methylcyclohexyl)acetamide, *cis*-
123	31.685	959838	332478	2.89	0.36	Acetamide, *N*-(4-hydroxycyclohexyl)-, *cis*-
124	31.755	1036345	432844	2.39	0.39	2-Amino-2-cyano-4-methylpentanethioamide
125	31.782	519951	452826	1.15	0.2	3,4-Altrosan
126	31.839	5081107	1301380	3.9	1.91	2-Propanone, 1-(4-hydroxy-3-methoxyphenyl)-
127	31.919	1405311	556255	2.53	0.53	1-(*p*-Toluidino)-1-deoxy-.beta.-d-idopyranose
128	31.977	1587430	573405	2.77	0.6	.beta.-D-Glucopyranose, 1,6-anhydro-

129	32.043	2105947	565041	3.73	0.79	Hexanoic acid, 6-bromo-
130	32.073	1381005	596863	2.31	0.52	Stevioside
131	32.145	3296028	708719	4.65	1.24	[2-[3-(2-Ureido-ethyl)-phenyl]-ethyl]-urea
132	32.242	2882114	793181	3.63	1.09	.beta.-D-Glucopyranose, 1,6-anhydro-
133	32.258	295923	747155	0.4	0.11	.alpha.-D-Galactopyranoside, methyl
134	32.281	2734383	753542	3.63	1.03	5-Methyl-6-[4-methoxyphenyl]-5,6-dihydrofuro[2,3-d]-2,4-3*H*-pyrimidinedi
135	32.348	2592862	693511	3.74	0.98	Hexanoic acid, 6-bromo-
136	32.399	1751793	767266	2.28	0.66	Nonanoic acid
137	32.482	2574178	813151	3.17	0.97	Silane, trimethyl(4-phenoxybutoxy)-
138	32.492	1706522	821290	2.08	0.64	2,3-Anhydro-d-mannosan
139	32.573	4005678	823188	4.87	1.51	.beta.-D-Glucopyranose, 1,6-anhydro-
140	32.642	6005262	868785	6.91	2.26	.beta.-D-Glucopyranose, 1,6-anhydro-
141	32.811	1477462	375696	3.93	0.56	4-(1-Hydroxyallyl)-2-methoxyphenol
142	33.033	692485	259983	2.66	0.26	1-(2-Hydroxy-4-methoxyphenyl)propan-1-one
143	33.168	254946	125127	2.04	0.1	Butyrovanillone
144	33.215	323768	117371	2.76	0.12	Diethyl Phthalate
145	33.648	235414	71535	3.29	0.09	Guanosine
146	33.895	76072	22533	3.38	0.03	Alachlor
147	34.266	323671	69506	4.66	0.12	1,4-epoxynaphthalene, 1,4-dihydro-1-methyl-
148	34.322	33882	29849	1.14	0.01	10-Acetoxy-2-hydroxy-1,2,6a,6b,9,9,12a-heptamethyl-1,3,4,5,6,6a,6b,7,8,8a,9,10,11,12,12a,12b,13,14b-octadecahydro-2*H*-picene-4a-
149	34.358	32071	11672	2.75	0.01	3,3,7-Trimethyl-oct-6-enoic acid, methyl ester
150	34.425	291841	32773	8.9	0.11	2-Butanone, 4-(4-hydroxy-3-methoxyphenyl)-
151	34.542	306805	116221	2.64	0.12	Propionic acid, 3-(allylthio)-, ethyl ester
152	34.63	2629525	575805	4.57	0.99	Benzenepropanol, 4-hydroxy-3-methoxy-
153	34.792	740054	194966	3.8	0.28	2-Naphthalenol, 3-methoxy-
154	34.838	20354	24413	0.83	0.01	4-Methoxyphenyl methyl carbinol
155	35.148	205242	49298	4.16	0.08	2-Propenthioamide, 2-cyano-3-(3-pyridylamino)-
156	36.353	506626	186801	2.71	0.19	Coniferyl aldehyde
157	36.827	93315	39914	2.34	0.04	1-Naphthol, 5,7-dimethyl-
158	37.09	574171	162655	3.53	0.22	2-Acetyl-1-tetralone
159	37.175	76903	43794	1.76	0.03	5-(2,4-Dimethyl-phenyl)-2H-pyrazol-3-ol
160	37.678	160075	65140	2.46	0.06	Benzenepropanol, 4-hydroxy-3-methoxy-
161	38.081	87932	42241	2.08	0.03	.alpha.-Corocalene
162	38.278	9644	12675	0.76	0	Propanoic acid, 2-methyl-, (dodecahydro-6a-hydroxy-9a-methyl-3-methylene-2,9-dioxoazuleno[4,5-b]furan-6-yl)methyl ester, [3aS-(
163	39.505	63504	30097	2.11	0.02	5*H*-Dibenzo[a,d]cyclohepten-5-ol, 10,11-dihydro-
164	39.999	134689	57001	2.36	0.05	5*H*-Dibenzo[a,d]cyclohepten-5-ol, 10,11-dihydro-
165	40.607	162314	68658	2.36	0.06	5-[*p*-Methoxyphenyl]-2,4-pentadienoic acid
166	42.254	277291	100132	2.77	0.1	Phenanthrene, 3,6-dimethyl-
167	44.458	285998	93750	3.05	0.11	9-Ethyl-10-methylanthracene
168	44.889	170037	58059	2.93	0.06	*n*-Pentadecanol
169	45.63	785121	318307	2.47	0.3	Retene
